# Caffeic Acid, Quercetin and 5-Fluorocytidine-Functionalized Au-Fe_3_O_4_ Nanoheterodimers for X-ray-Triggered Drug Delivery in Breast Tumor Spheroids

**DOI:** 10.3390/nano11051167

**Published:** 2021-04-29

**Authors:** Stefanie Klein, Luitpold V. R. Distel, Winfried Neuhuber, Carola Kryschi

**Affiliations:** 1 Department of Chemistry and Pharmacy, Physical Chemistry I and ICMM, Friedrich-Alexander University of Erlangen-Nuremberg, Egerlandstr. 3, D-91058 Erlangen, Germany; carola.kryschi@fau.de; 2 Department of Radiation Oncology, Friedrich-Alexander University of Erlangen-Nuremberg, Universitätsstr. 27, D-91054 Erlangen, Germany; luitpold.distel@fau.de; 3 Institute of Anatomy, Chair of Anatomy and Cell Biology, Friedrich Alexander University Erlangen-Nuremberg, Krankenhausstr. 9, D-91054 Erlangen, Germany; winfried.neuhuber@fau.de

**Keywords:** multicellular tumor spheroid, Au-Fe_3_O_4_ nanoheterodimers, radiotherapy, chemotherapy, X-ray-triggered drug delivery

## Abstract

Au-Fe_3_O_4_ nanoheterodimers (NHD) were functionalized with the natural and synthetic anticancer drugs caffeic acid (CA), quercetin (Q) and 5-fluorocytidine (5FC). Their X-radiation dose-enhancing potential and chemotherapeutic efficacy for bimodal cancer therapy were investigated by designing multicellular tumor spheroids (MCTS) to in vitro avascular tumor models. MCTS were grown from the breast cancer cell lines MCF-7, MDA-MB-231, and MCF-10A. The MCF-7, MDA-MB-231 and MCF-10A MCTS were incubated with NHD-CA, NHD-Q, or NHD-5FC and then exposed to fractionated X-radiation comprising either a single 10 Gy dose, 2 daily single 5 Gy doses or 5 daily single 2 Gy doses. The NHD-CA, NHD-Q, and NHD-5FC affected the growth of X-ray irradiated and non-irradiated MCTS in a different manner. The impact of the NHDs on the glycolytic metabolism due to oxygen deprivation inside MCTS was assessed by measuring lactate secretion and glucose uptake by the MCTS. The NHD-CA and NHD-Q were found to act as X-radiation dose agents in MCF-7 MCTS and MDA-MB-231 MCTS and served as radioprotector in MCF-10A MCTS. X-ray triggered release of CA and Q inhibited lactate secretion and thereupon disturbed glycolytic reprogramming, whereas 5FC exerted their cytotoxic effects on both, healthy and tumor cells, after their release into the cytosol.

## 1. Introduction

Breast cancer is the most common cancer in women with over 2 million new cases in 2018 according to the World Cancer Research Fund International. In order to avoid mastectomy, breast-conserving surgery is performed first, and followed by adjuvant whole-breast radiotherapy [1,2]. A typical radiotherapy plan includes daily fractions of 2 Gy, which are administered five times per week, until a maximum total dose of 50 Gy is reached [1,2,3]. Administration of the radiation dose in smaller fractions enables the reoxygenation of hypoxic cells, redistribution of cells through the cell cycle and repair and repopulation of normal tissue [3,4]. However, major obstacles for successful radiotherapy are the intrinsic radiation resistance of cell subpopulations of the tumor and the radiation resistance acquired during therapy. Cellular hypoxia, tumor niches and microenvironment as well as apoptosis and autophagy are key determinants for cancer radio-resistance [5,6].

Monolayer cell cultures are extensively used in cancer research because of their easy handling and high throughput rates. Despite these advantages, 2D cell cultures lack cell heterogeneity, cell-to-cell communication, metabolic gradients, and the microenvironment of real tumor tissues. In contrast to cell monolayers, tumor tissue comprises hypoxic cells and thus, is less sensitive to low-dosage X-ray irradiation. In the 3D cellular network of tumors, the oxygen, nutrients and pH gradients and metabolite exchange between adjacent cells maintain tumor progression and metastatic spread [7,8,9]. Multicellular tumor spheroids (MCTS) are self-assembled aggregates of cancer cells which mimic the 3D cellular architecture and pathophysiological gradients of avascular tumors and metastases and thus close the gap between monolayer cell cultures and animal studies [7,8,9,10,11,12]. Analogously, the penetration of oxygen, nutrients, and metabolites into MCTS is limited to depths smaller than 200 µm. Thus, sufficiently large MCTS (~400–500 μm diameter) possess a layered cellular structure: the necrotic core is surrounded by hypoxic (quiescent) cells and proliferating cells form the outer shell. Since hypoxic cells are chemo- and radio-resistant and undergo glycolytic metabolism, MCTS resemble essential in vivo features of tumors, and thus serve as adequate in vitro tumor models for the validation of novel multimodal anticancer therapeutics [7,10,11,12].

The X-ray sensitivity of tumors depends on a broad variety of parameters such as heterogeneity and density, pH and oxygen gradient, DNA repair and apoptosis induction mechanisms [13]. Quiescent and hypoxic cells are radioresistant due to their higher repair rate of potentially lethal damage [13,14,15,16]. X-ray-induced DNA damage in tumor cells is predominantly caused by reactive oxygen species (ROS). The efficiency of ROS formation depends on the oxygen level in cytoplasm. Hence hypoxic cell populations substantially contribute to the ineffectiveness of radiation therapy [14,17,18]. Since MCTS comprise hypoxic cells, they represent an adequate tumor model for the testing and first validation of anticancer and radio-enhancing agents for the fractionated clinical radiotherapy [13,19]. 

In former studies on monolayer cell cultures [20,21,22], we could prove that Au-Fe_3_O_4_ nanoheterodimers (NHDs) performed as excellent X-radiation dose enhancing agents in tumor cells. Both, the gold and Fe_3_O_4_ components were shown to contribute to the enhancement of the X-ray efficacy in complementary ways. X-ray irradiated gold nanoparticles, as consisting of high-Z atoms, emit secondary X-rays, Auger- or photoelectrons [23,24,25,26], whereas the surface Fe^2+^ cations catalyzed the Fenton reaction so that highly toxic ^•^OH radicals are generated [27,28,29]. We reported that caffeic-acid functionalized Au-Fe_3_O_4_ NHDs perform as radiosensitizers in breast cancer cells but acted as radioprotectors in healthy breast epithelial cells in 2D cell cultures [21]. 

In this contribution, we functionalized Au-Fe_3_O_4_ NHDs with the natural and synthetic anticancer drugs caffeic acid, quercetin and 5-fluorocytidine and investigated their X-radiation dose enhancing and chemotherapeutic impact on MCTS. The MCTS under study were grown from the breast cancer cell lines MCF-7 and MDA-MB-231 as well as from the non-tumorigenic breast epithelial MCF-10A cells. 5-Fluorocytidine is a modified prodrug of the anticancer drug 5-fluorouracil (5FU) used to treat breast cancer [30,31]. 5FU interferes with the nucleoside metabolism and is incorporated in the RNA and DNA strands which leads to cell death [32,33,34] Caffeic acid and quercetin are flavonoids with antioxidant, anticancer and antimetastatic effects [35,36,37,38]. In this study, we show that surface-bound caffeic acid and quercetin act as X-radiation-enhancing agents in MCF-7 based MCTS, but they protect MCF-10 A MCTS against X-rays. These results are consistent with results obtained from analogous experiments performed on the respective 2D cell cultures and are ascribed to the particular cell-specific redoxactive properties of caffeic acid and quercetin [21]. In comparison, the effectiveness of 5-fluorocytidine-functionalized Au-Fe_3_O_4_ NHDs in X-ray irradiated MCTS did not only consist in enhanced ROS production but also in the X-ray-induced release of 5-fluorocytidine, which damages tumor cells as an anticancer drug [32,33]. In contrast, the non-tumorigenic MCF-10A MCTS loaded with the 5-fluorocytidine-functionalized Au-Fe_3_O_4_ NHDs survived fractionated X-radiation treatment with 5 single doses of 2 Gy. 

## 2. Materials and Methods

### 2.1. Chemicals

Caffeic acid (98%), methanol (≥99.8%), trypan blue (≥80%) and glutaraldehyde (25%) were purchased from Carl Roth GmbH + Co. KG, Karlsruhe, Germany. Penicillin-streptomycin-solution, sodium pyruvate, phosphate-buffered saline (PBS), non-essential amino acids, trypsin/EDTA (ethylenediaminetetraacetic acid), di-sodium EDTA (99%), L-lactic dehydrogenase from rabbit muscle (L-LDH), lithium L-lactate (98%), β-NAD (96,5%), β-NADPH (≥97%), glycine (≥99%), hydrazine sulfate (≥99%), agarose (BioReagent), glutathione reductase from baker’s yeast, Tris-HCl (99%), 5-fluorocytidine (97%), quercetin (≥95%), HCl (37%), potassium hexacyanoferrate(II) (ACS reagent), Na_2_CO_3_ (p.a.), DL-tartaric acid (>99%), NaOH (97%), 2′,7′-dichlorofluorescein diacetate (95%), 5,5′-dithiobis(2-nitrobenzoic acid) (98%), 5-sulfosalicylic acid (≥99%), reduced and oxidized glutathione (98%), D-(+)-glucose, MgSO_4_ anhydrous (≥99.5%), insulin solution human, hydrocortisone (≥98%), cholera toxin from Vibrio cholera (95%), triethanolamine (99%) and 2-vinylpyrindine (97%) were purchased from Sigma-Aldrich Inc, St. Louis, MO, USA. Fetal calf serum (FCS), GlutaMAX Supplement, Dulbecco’s modified Eagle medium (DMEM), DMEM/F12 and horse serum were bought from Thermo Fischer Scientific Inc, Waltham, MA, USA and human epidermal growth factor from Pepro Tech Inc., Rocky Hill, NJ, USA KCl (99.5%), NaCl (99.5%) and MgCl_2_ anhydrous (98%) were ordered from Fluka and NaHCO_3_ (99.5%), CaCl_2_ anhydrous (p.a.), KH_2_PO_4_ (p.a.), K_2_HPO_4_ (99%), CuSO_4_ anhydrous (99%), molybidic acid (85%) and sodium tungstate (99%) from Merck KGaA, Darmstadt, Germany. Ultrapure water with a conductivity of 18 (MΩ)^−1^cm^−1^ was used during the experiments.

### 2.2. Instrumentation 

Fourier transform infrared (FTIR) spectroscopy was performed on a Bruker Tensor 27 spectrometer (Bruker, Billerica, MA, USA). The MCTS were imaged under an inverted optical microscope (Wilvort S, Helmut Hund GmbH, Wetzlar, Germany) equipped with a digital camera. Transmission electron microscopy (TEM) images were taken with a LEO 906 TEM (Carl Zeiss AG, Oberkochen, Germany). The cell assays were conducted using a Synergy HT microplate reader (BioTek Inc., Winooski, VT, USA). The X-ray irradiation of the MCTS was performed using a X-ray tube with an average energy of 34 keV (Comet MXR 160/0.4-3.0, COMET AG, Flamatt, Switzerland).

### 2.3. Synthesis of the Au-Fe_3_O_4_ Nanoheterodimers

The synthesis procedure was described in detail in our previous publication [20].

### 2.4. Ligand Exchange Procedure 

Biocompatibility and water dispersibility of the Au-Fe_3_O_4_ nanoheterodimers were obtained by partial exchange of the oleylamine and oleic acid ligands by caffeic acid, quercetin or 5-fluorocytidine. Details of the procedure were given by S. Klein et al. [21].

### 2.5. Multicellular Tumor Spheroids (MCTS)

MCF-7, MDA-MB-231 or MCF-10 A MCTS were prepared by following the procedure by J. Friedrich et al. [39]. In short, 96 well plates were covered with agarose gel (1.5%) for low attachment. The NHD stock solutions of 1 mg mL^−1^ were autoclaved at 120 °C, 2 bar for 20 min. MCF-7 cells (20,000 cells/well) and MDA-MB-231 cells (15,000 cells/well) were grown in DMEM medium supplemented with 10% FCS, 1 mM sodium pyruvate, 100 U/mL penicillin, 100 μg/mL streptomycin, 2 mM L-glutamine and 1% non-essential amino acids. 15,000 MCF-10A cells per well were kept in DMEM/F12 medium with 5% horse serum, 20 ng/mL EGF (epidermal growth factor), 0.5 mg/mL hydrocortisone, 100 ng/mL cholera toxin, 10 μg/mL insulin, 10 mM HEPES (4-(2-hydroxyethyl)-1-piperazineethanesulfonic acid) buffer and 100 U/mL penicillin, 100 μg/mL streptomycin. All cells were cultivated for 7 days until a single spheroid was formed in each well, whereas the medium was changed every second day. Afterwards, the MCTS were treated for 72 h with 50 μg mL^−1^ NHD-CA, NHD-Q or NHD-5FC. The growth curves of NHD loaded and unloaded MCTS were measured subsequently to incubation of the MCTS in medium without and with NHDs for 72 h. The MCTS were either irradiated with a single dose of 10 Gy, two fractions of 5 Gy, five fractions of 2 Gy or left untreated. Size and morphology of the MCTS were determined under an optical microscope at day 0, 1, 3, 5 and 7 after X-ray irradiation. The volume of the MCTS were calculated as described by D. Khaitan et al. [18]. The tumor growth curves were fitted according to mathematical models [40,41].

### 2.6. Preparation for Transmission Electron Microscopy (TEM) Images 

The MCTS were grown and incubated with the NHDs as described. 40–50 MCTS were transferred into a 2 mL reaction tube. The MCTS were centrifuged at 200× *g* for 5 min and washed two times with PBS. The MCTS were fixed with 2.5% glutaraldehyde in PBS at 4 °C for 24 h and postfixed in 1% osmium tetroxide and 3% potassium ferricyanide at room temperature. The MCTS were dehydrated through grade alcohols and embedded in Epon. Non-contrasted and with uranyl acetate-contrasted silver-gray ultrathin sections were imaged.

### 2.7. Determination of Glucose Consumption and Lactate Secretion

We transferred 20 MCTS to a 2 mL reaction tube and washed them twice with PBS. The MCTS were incubated in a buffer solution (0.1 mg/mL glucose, 1.3 mM CaCl_2_, 0.5 mM MgCl_2_, 0.4 mM MgSO_4_, 5.3 mM KCl, 0.4 mM KH_2_PO_4_, 4.2 mM NaHCO_3_, 138 mM NaCl, 0.3 mM Na_2_HPO_4_). After 2 h and 4 h a sample of the surrounding buffer was taken. The amount of the remaining glucose and the produced lactate was determined.

#### 2.7.1. Lactate Assay

We mixed 20 μL of the samples with 250 μL 0.5 M glycine and 0.4 M hydrazine buffer (pH 9), 25 μL NAD solution (17 mg/mL) and 2.5 μL L-LDH (300 units/mL). After 1 h of incubation at 25 °C, the fluorescence was excited at 340 nm and the fluorescence intensity was measured at 460 nm. The L-lactate concentration of the samples was calibrated with L-lactate solutions with concentrations from 0 to 2 mM and were normalized to the number of spheroids and incubation time. 

#### 2.7.2. Estimation of Sugar by Folin and Wu Method

We mixed 100 μL of the sample with 100 μL of an alkaline copper solution and kept in a boiling water bath for 8 min. After cooling to room temperature, 100 μL of a phosphomolybidic acid solution were added. After the color reaction has taken place (within 10 min), the absorbance at 420 nm was measured. The glucose concentration was calibrated by means of glucose solutions with concentrations in the range of 0–100 μg/mL and normalized using the number of MCTS and a constant incubation time.

### 2.8. Fractionating of the MCTS

The MCTS were transferred to a 6-wellplate and incubated with 0.05% trypsin/EDTA solution for 5 min for the MCF-7 MCTS or 10 min for the MDA-MB-231 and MCF-10 A MCTS. By gently shaking the surface, cells were separated and the floating cells with the supernatant were removed. The cells were collected by centrifugation (200× *g*, 5 min) and resuspended in cell culture medium. These steps were repeated two times and the cells were defined as the surface region. The remains of the MCTS were incubated with 0.25% trypsin/EDTA solution which provided the disaggregation of the cells. The floating single cells were separated from the bigger cell aggregates. The single cells were the necrotic ones of the core. The cell aggregates were separated with Trypsin/EDTA and represent the quiescent region of the MCTS.

### 2.9. Cell Viability

The cell viability of the separated cells was determined with the trypan blue staining. Equal volumes of cell suspension and trypan blue solution (0.4% in PBS) were mixed and counted with a hemocytometer.

### 2.10. Intracellular Iron Content by Prussian Blue Reaction

To determine the intracellular iron concentration of the separated MCTS regions, the iron oxide part of the various NHDs were dissolved. After cell counting, the cells and intracellular NHDs were digested with 37% HCl for 24 h at 37 °C followed by 4 h at 80 °C. As an iron-dosing method, the Prussian blue reaction was used [42,43]. The samples were mixed with 5% potassium ferrocyanide solution and the absorbance at 630 nm was measured. The iron concentration of the cell samples was calibrated with FeCl_3_ standards (0–50 μg mL^−1^) and all samples were normalized to the cell number.

### 2.11. Intracellular Reactive Oxygen Species (ROS) Concentration

After separation of the MCTS, the cells were resuspended in PBS buffer and 10,000 cells of each sample was filled in a 96 well plate. 2′,7′-dichlorofluorescein diacetate (10 mM stock solution in dimethyl sulfoxide (DMSO)) was added to each well to reach a final concentration of 100 μM/well. The plates were put in an incubator for 30 min. Afterwards, the fluorescence was measured at 485 nm excitation and 528 nm emission. 

## 3. Results and Discussion

### 3.1. Characterization of the Functionalized Au-Fe_3_O_4_ Nanoheterodimers (NHDs)

**Figure 1:**
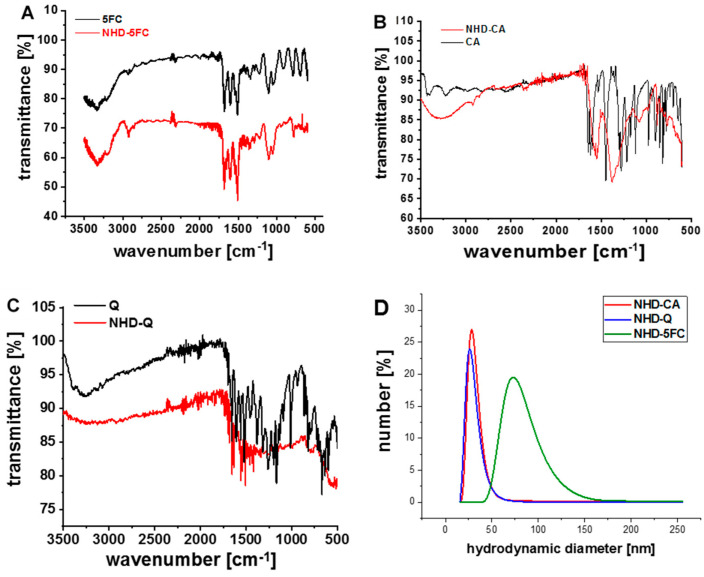
Fourier transform infrared (FTIR) transmittance spectra of 5-fluorocytidine (5FC) and nanoheterodimer NHD-5FC (**A**); caffeic acid (CA) and NHD-CA (**B**); quercetin (Q) and NHD-Q (**C**), Dynamic light scattering measurements of the various NHDs in phosphate-buffered saline (PBS) (**D**).

Snowman-shaped Au-Fe_3_O_4_ NHDs with sizes around 12 nm were synthesized by means of a two-step thermal decomposition technique [20]. Biocompatible, functionalized Au-Fe_3_O_4_ NHDs were obtained by exchanging the oleic acid and oleylamine ligands with caffeic acid (CA), quercetin (Q) and 5-fluorocytidine (5FC). FTIR transmittance spectra were recorded to examine the surface binding of 5FC, CA and Q (Figure 1A–C). The results obtained from DLS (dynamic light scattering) measurements of the NHD-CA and NHD-Q reveal small hydrodynamic sizes of 30 nm with a narrow size distribution (Figure 1D). The DLS curves of the NHD-5FC arose from a broader size distribution indicating the formation of larger agglomerates. This is presumably due to weak surface binding of 5FC by hydrophobic interactions which is suggested by the FTIR spectrum. Zeta potential measurements of the NHD-5FC, NHD-Q and NHD-CA result in negative surface charge values with−38.8 ± 3.20 mV, −64.7 ± 5.06 mV and −47.7 ± 3.74 mV, respectively. Moreover, we determined the respective drug-loading content (DLC) from the ultraviolet–visible (UV-Vis) absorption spectra of the NHD-5FC, NHD-Q and NHD-CA dispersed in ethanol at a concentration of 40 µg/mL (SI Figure S1). The obtained DLC values in wt.% for NHD-Q, NHD-CA and NHD-5FC are 4.5 ± 0.6%, 3.1 ± 0.7% and 3.4 ± 1.2%. 

### 3.2. Growth of the MCTS without Irradiation

#### 3.2.1. MCF-7 MCTS

**Figure 2:**
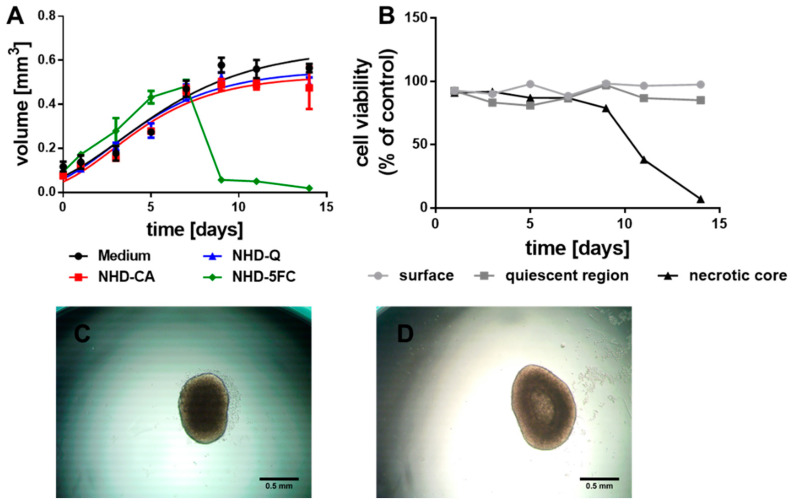
Growth curves of the MCF-7 MCTS (**A**) loaded with Q-, CA- and 5FC-functionalized Au-Fe_3_O_4_ NHDs (i.e., NHD-CA, NHD-Q, NHD-5FC); the cell viability of the surface, quiescent and necrotic zones of untreated MCTS (**B**), micrographs of a MCTS after 5 (**C**) and 9 (**D**) days growth in culture medium, n = 12.

**Figure 3:**
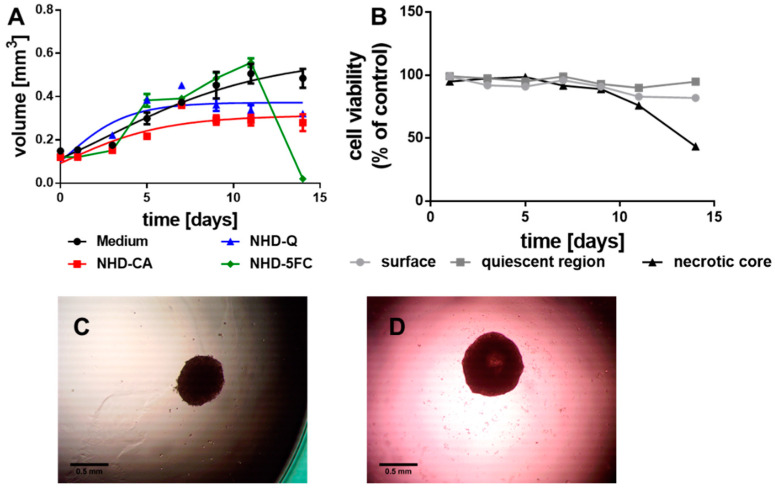
Growth curves of the MDA-MB-231 MCTS (**A**) loaded with NHD-CA, NHD-Q and NHD-5FC; the cell viability of the surface, quiescent and necrotic zones of untreated MCTS (**B**), micrographs of a MCTS after 5 (**C**) and 11 (**D**) days growth in culture medium, n = 12.

To assess the impact of CA-, Q- and 5FC-functionalized Au-Fe_3_O_4_ NHDs on breast tumor tissue, we used MCTS as in vitro tumor models. MCTS were grown from MCF-7 and MDA-MB-231 cells (Figure 2 and Figure 3). MCF-7 cells are estrogen and progesterone receptor positive cells with low metastatic potential, which belong to the luminal A subtype of breast cancer [44,45]. The triple negative MDA-MB-231 breast adenocarcinoma cells lack the estrogen and progesterone receptor and HER-2 (human epidermal growth factor receptor 2) amplification [46]. These highly metastatic cells belong to the basal subtype of breast cancer [45]. The growth curve of the MCF-7 MCTS follows a hyperbolic function that reflects a saturation kinetics (Figure 2A, black line). Incubation of the MCF-7 MCTS with CA- (NHD-CA, red line) and Q-functionalized Au-Fe_3_O_4_ NHDs (NHD-Q, blue line) decreased the growth curve maximum by 13% and 10%, respectively (Figure 2A, Figure S2). In comparison, the MCF-7 MCTS response on 5FC-functionalized Au-Fe_3_O_4_ NHDs (NHD-5FC, green line) consisted in a faster growth behavior within the first five days followed by a steep decay of the volume due to spheroid disaggregation (Figure 2A, Figure S2). The growth kinetics of the differently stained MCTS are consistent with the results obtained from cell viability assays (Figure S3). MCTS larger than 500 µm in diameter were sequentially trypsinized to isolate-proliferating cells (surface layer), hypoxic (quiescent) cells and necrotic cells (necrotic core). While the NHD-Q and NHD-CA only slightly decreased the cell viability, the NHD-5FC were obviously cytotoxic for the proliferating and hypoxic tumor cells. This is explained with the release of the anticancer drug 5FC into the cytoplasm. The growth of MCTS is associated with the development of an oxygen gradient, which affects the viability of tumor cells in dependence on their location in the necrotic, hypoxic (quiescent), or proliferating region. The viability of necrotic, hypoxic, and proliferating cells of untreated MCTS was measured as function of the growth time (Figure 2B). As expected, the viability of proliferating and hypoxic tumor cells did not change over 14 days of growth, while cells in the MCTS core deceased after day 7 (Figure 2B). This result was confirmed by the time-evolution of the MCTS features illustrated in the micrographs (Figure 2C,D). At day 5 of MCTS growth the core appeared to be dark and thus compact. The color change of the core from dark to bright indicates its dissolution.

#### 3.2.2. MDA-MB-231 MCTS

In comparison with the MCF-7 MCTS, the MDA-MB-231-derived tumor spheroids grew slower (Figure 3A, black line) which we explain with the basal nature of this breast tumor cell line. These MCTS, when incubated with the NHD-CA, NHD-Q, and NHD-5FC, reached a significantly smaller volume after 14 days of growth (Figure 3A, images Figure S4). The smaller final MCTS volumes correlate with the results obtained from the cell viability assay (Figure S5). The Au-Fe_3_O_4_-NHDs reduced most severely the viability of proliferating cells which disrupted the MCTS growth. In contrast, the proliferating and hypoxic cells of the untreated MCTS did not change their viability over 14 days of growth, whereas the cells in the core began to die after 9 days when the MCTS had reached 1 mm in size (Figure 3B). The micrograph of the MDA-MB-231 MCTS taken at the 5th day of growth exhibited an unchanged compact core (Figure 3C). After 11 days of growth, the core became bright, which indicates cell death (Figure 3D). 

#### 3.2.3. MCF-10A Multicellular Spheroids (MCS)

**Figure 4:**
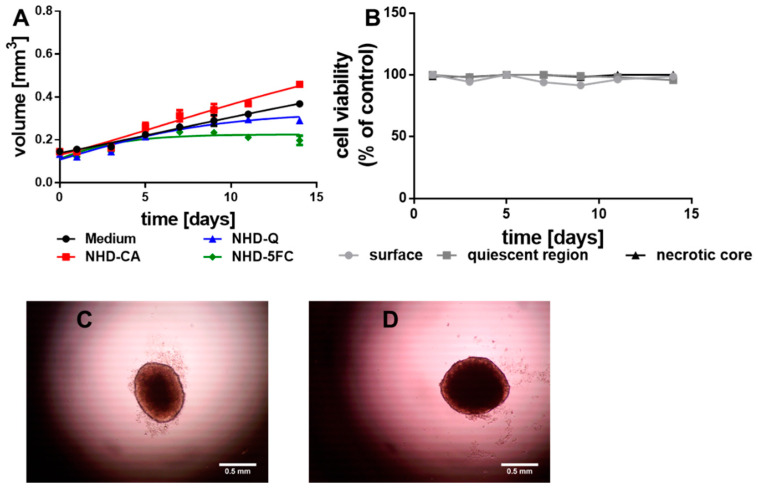
Growth curves of the MCF-10 A multicellular spheroids (MCS) (**A**) loaded with NHD-Q, NHD-CA, and NHD-5FC; the cell viability of the surface, quiescent and necrotic zones of untreated MCS (**B**), micrographs of a MCS after 5 (**C**) and 14 (**D**) days growth in culture medium, n = 12.

To survey the cytotoxic impact of the NHD-CA, NHD-Q, and NHD-5FC on healthy tissue, multicellular spheroids (MCS) were grown from non-tumorigenic breast epithelial cells MCF-10 A. The MCF-10 A MCS grew very slowly and reached a diameter of 1 mm after 14 days (Figure 4A, black line). Incubation with the NHD-CA, NHD-Q, and NHD-5FC significantly changed the growth kinetics and, thereby, the final diameter. Intracellular NHD-5FC stopped the MCTS growth after the 7th day, while NHD-CA-loaded MCF-10A MCS showed a faster growth kinetics (Figure 4A, Figure S6). Cell viability studies on NHD-loaded MCF-10A MCS demonstrated that the NHD-CA and NHD-Q are biocompatible, while the NHD-5FC are cytotoxic (Figure S7). In contrast to MCTS grown from breast tumor cells, the viability of the proliferating (surface), hypoxic (quiescent) and necrotic cells of untreated MCF-10 A MCS were nearly constant over the growth period of 14 days (Figure 4B). The microscopy images of MCF-10 A MCTS recorded at day 5 and day 14 demonstrate that the overall cell density did not change during the MCS growth (Figure 4C,D). 

### 3.3. Metabolism of the Spheroids

**Figure 5:**
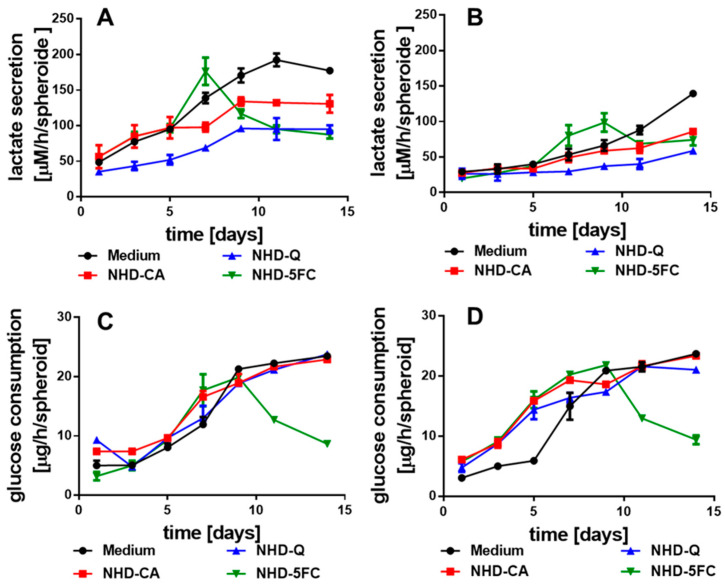
Lactate secretion of the MCF-7 (**A**) and MDA-MB-231 (**B**) MCTS in medium with and without CA-, Q-, and 5FC-functionalized Au-Fe_3_O_4_ NHDs, glucose consumption of the MCF-7 (**C**) and MDA-MB-231 (**D**) MCTS with and without NHDs, n = 12.

The emergence of hypoxia during MCTS growth provides the change of the metabolic pathway to anaerobic glycolysis. As an immediate consequence, the hypoxic tumor cells consume glucose to produce lactate that is secreted by the monocarboxylate transporter MCT4. The lactate efflux induces metabolic reprogramming of adjacent oxidative cells which ingest lactate to fuel the TCA cycle [47,48,49]. Hence, glucose consumption and lactate secretion scale with the number of hypoxic cells in MCTS. For MCF-7 and MDA-MB-231 MCTS, grown in medium, the lactate secretion increased after the first day (Figure 5A), and after the third day (Figure 5B), respectively. In contrast, the lactate secretion inside MCF-10 A MCS remained unchanged over the whole growth period (Figure S8A). CA and Q were reported to inhibit the MCT4 [50,51,52]. This is consistent with our observations. MCF-7-MCTS loaded with the NHD-CA showed a decreased lactate secretion (after 14 days) that was 23% smaller than the lactate secretion of the untreated MCTS, whereas the NHD-Q decreased lactate secretion by 45% (Figure 5A). In case of the MDA-MB-231 MCTS the NHD-CA and NHD-Q lowered the lactate secretion by 43 and 70%, respectively (Figure 5B). Inhibition of the MCT4 blocks the lactate efflux and increases the intracellular lactate level, which could also be demonstrated for the NHD-Ca and NHD-Q (Figure S9). 5FC acts on the glycolytic metabolism in a different manner than CA and Q. The NHD-5FC apparently reduced the lactate secretion after the 7th day (Figure 5A,B). The drastic decrease in lactate concentration is due to 5FC-induced damaging of the MCF-7 and MDA-MB-231 MCTS which is also reflected by the corresponding MCTS growth curves (Figure 3A,B). As expected, the NHD-CA, NHD-CA, and NHD-5FC exerted a vanishingly small impact on the lactate secretion in MCF-10A MCS (Figure S8A). 

MCF-7 and MDA-MB-231 breast cancer cells exhibited increased expression of the glucose transporter 1 (GLUT1) providing glucose uptake [53]. The potential inhibitory effect of the NHD-CA, NHD-Q, and NHD-5FC on the GLUT1 in MCF-7, MDA-MB-231 and MCF-10A derived MCTS was studied by measuring the glucose consumption during the growth of the respective MCTS (Figure 5C,D, Figure S8B). In MCF-7 and MDA-MB-231 MCTS the NHD-CA and NHD-Q inhibited the glucose uptake only insignificantly. In the case of the NHD-5FC, the decrease in glucose uptake after day 7 correlates with the 5FC-induced eradication of breast tumor cells (Figure 5C,D). Similar results were obtained for the MCF-10A MCS (Figure S8B). In any case, the increased glucose uptake and lactate secretion of MCF-7 and MDA-MB-231 MCTS during their growth indicates the emergence of hypoxia and the associated glycolytic metabolism. In contrast, the MCF-10A MCS showed only a small increase in glucose uptake and lactate secretion during the 14-day growth period. 

### 3.4. Internalization of the Functionalized Au-Fe_3_O_4_ NHDs 

**Figure 6:**
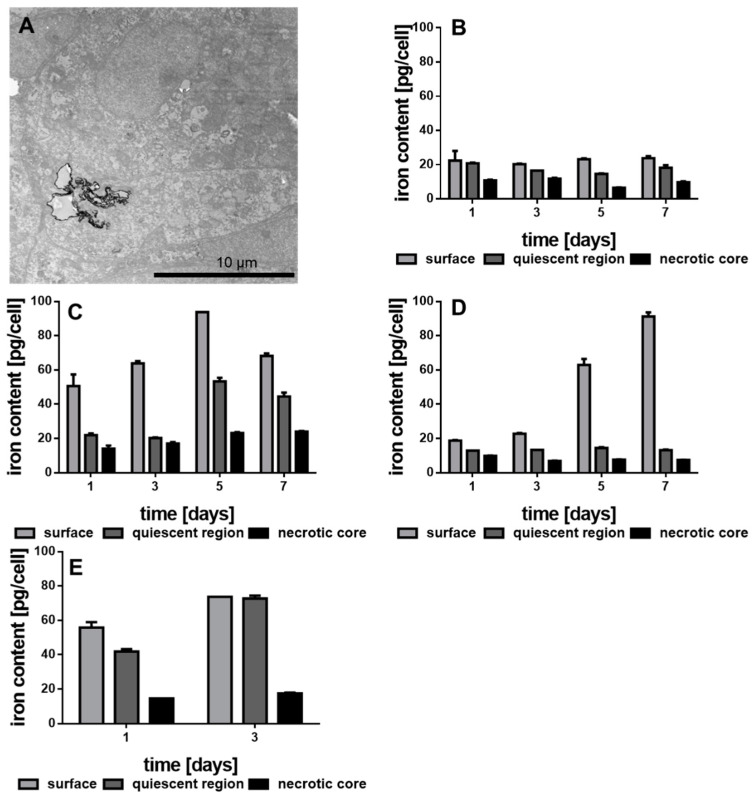
Transmission electron microscopy (TEM) image of MDA-MB-231 MCTS treated with NHD-CA (**A**), iron content of the separated MDA-MB-231 MCTS regions cultivated in medium (**B**) or in medium with NHD-CA (**C**), NHD-Q (**D**) and NHD-5FC (**E**), n = 12.

Both, growth kinetics and lactate secretion of the different MCTS should depend on the concentration of internalized Au-Fe_3_O_4_ NHDs. Figure 6A shows a TEM image of the central section of one MDA-MBA-231 MCTS incubated with NHD-CA. The Au-Fe_3_O_4_ NHDs are visible as dark clusters. To provide a quantitative measure of internalized NHDs, we determined the intracellular iron content of the three different zones (necrotic, quiescent, and surface) of the MDA-MB-231 MCTS grown in culture medium without and with the differently functionalized NHDs (Figure 6B–E). As expected, the iron content of proliferating (surface), hypoxic (quiescent) and necrotic MDA-MB-231 cells does not really change over the 7-day growth period of the MCTS grown in NHD-free medium (Figure 6B). In contrast, the Fe content of MCTS containing NHD-CA reached its largest concentration (94 pg/cell) in proliferating cells at day 5, whereas the maximum values of the Fe content in hypoxic and necrotic cells are 54 and 24 pg/cell, respectively (Figure 6C). These results demonstrate the preferential uptake of NHD-CA by proliferating surface cells and, in addition, prove that the NHD-CA invaded the necrotic core. In case of MCTS incubated with NHD-Q, the Fe content in proliferating cells is maximal at day 7 (90 ng/cell), whereas the Fe content in hypoxic and necrotic cells is equal to the values of MCTS incubated in NHD-free medium (Figure 6D). Apparently, the NHD-Q were internalized only by the proliferating cells but did not invade the hypoxic and necrotic cells. The distribution of the NHD-5FC throughout the MDA-MB-231 MCTS is depicted in Figure 6E. Since intracellular 5FC damages tumor cells, the cellular uptake of the NHD-5FC by proliferating, hypoxic and necrotic cells could be followed over 3 days only. The maximum values of the Fe content in proliferating and hypoxic cells are around 75% at day 3, which demonstrates efficient penetration of the NHDs into the MCTS and explains the loss of MCTS integrity after 5 days growth. A similar cellular uptake behavior of the NHD-CA, NHD-Q, and NHD-5FC was observed for the proliferating, hypoxic and necrotic MCF-7 tumor cells and MCF-10A epithelial cells (Figures S10 and S11).

### 3.5. Irradiated MCTS without Au-Fe_3_O_4_ NHDs 

**Figure 7:**
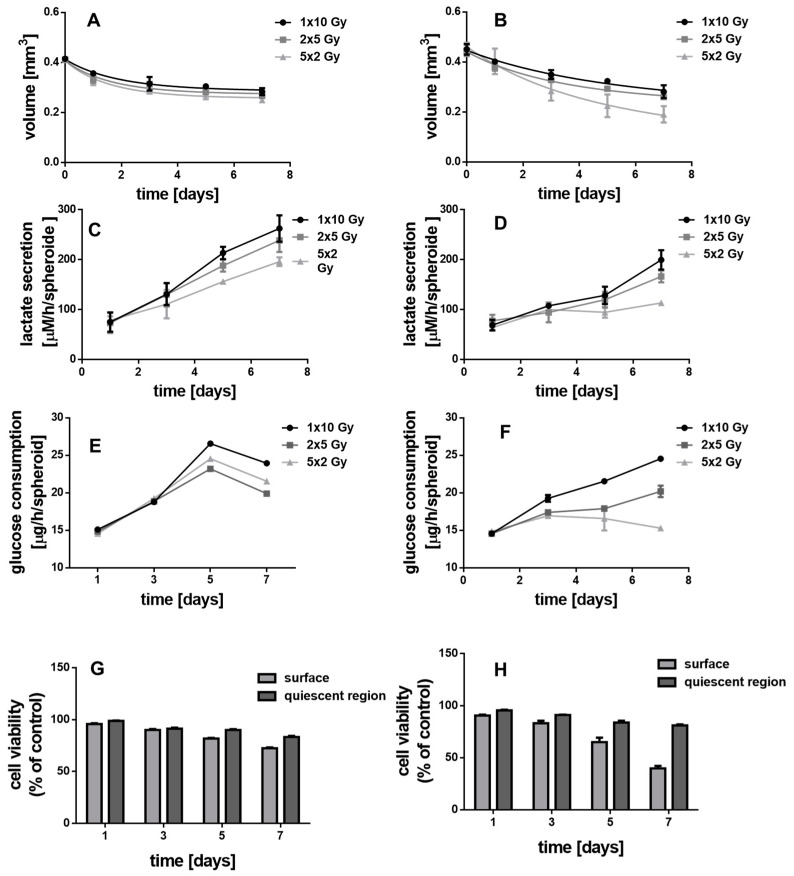
The impact of fractionated X-ray irradiation on the MCF-7 (**A**,**C**,**E**,**G**) and MDA-MB-231 (**B**,**D**,**F**,**H**) MCTS on the MCTS growth (**A**,**B**), lactate secretion (**C**,**D**), glucose consumption (**E**,**F**) and cell viability of the proliferating surface and quiescent region (**G**,**H**), n = 12.

The X-ray enhancing potential of the NHD-CA, NHD-Q, and NHD-5FC inside breast tumor and epithelial cell spheroids was investigated by exposing the MCF-7 and MDA-MB-231 MCTS to a single dose of 10 Gy or to daily fractions comprising 2 daily single 5 Gy doses or 5 daily single 2 Gy doses. The treatment of the MCTS with a single 10 Gy dose reduced the MCF-7 and MDA-MB-231 MCTS volumes by 20–25% 7 days after X-ray irradiation. The fractionated X-radiation treatment hardly reduced the MCF-7 MCTS volumes (Figure 7A,B). The impact of fractionated X-radiation doses on the volumes of the MDA-MB-231 MCTS is significantly larger: the MCTS volume decreased from 0.45 mm^3^ to 0.2 mm^3^ 7 days after fractionated X-radiation treatment with 5 single doses of 2 Gy (Figure 7B). Obviously, the fractionated X-radiation treatment of MCTS is more efficient for the destruction of tumor cells than the administration of one single dose of 10 Gy. Distributing a 10 Gy dose over 5 days allows hypoxic cells to recover by reoxygenation between administration of the 2 Gy doses, which makes X-ray irradiation-induced damage more efficient [54,55]. 

The effect of fractionated X-ray irradiation on glycolysis metabolism was studied by measuring lactate secretion and glucose uptake of untreated MCF-7 and MDA-MB-231 MCTS over 7 days of spheroid growth (Figure 7C–F). In comparison to the treatment with a single dose of 10 Gy the growth curves of both lactate secretion and glucose uptake in MCF-7 and MDA-MB-231 MCTS were reduced in their gradient by administration of 2 single 5 Gy doses and at most, by administration of 5 single 2 Gy doses. This is in line with the observed X-ray irradiation-induced decrease of the MCTS volume over 7 days. Cell viability studies of the surface and quiescent layers of the MCF-7 and MDA-MB-231 MCTS treated with a single 10 Gy dose, 2 single 5 Gy doses or 5 single 2 Gy doses over 7 days demonstrated that the proliferating cells in the surface layer suffered greater radiation damage than the deeper located hypoxic cells in the quiescent zone of the respective MCTS (Figure 7G,H). Interestingly, the damage of the normoxic tumor cells of MCF-7 MCTS by X-rays is much lower than that of MDA-MB-231 MCTS. The cells in the quiescent layer of both, MCF-7 and MDA-MB-231 MCTS, were only slightly reduced in viability which is due the radioresistance of hypoxic cells. Nevertheless, fractionated X-ray irradiation with 5 single 2-Gy doses is most efficient for tumor cell damage. This is explained by the 24 h interval between the administration of single doses, which allows reoxygenation of the surviving tumor cells and thereupon increases their radiosensitivity [54,55] In a nutshell, X-radiation treatment of MCF-7 and MDA-MB-231 MCTS with one single 10 Gy dose, 2 single 5 Gy or 5 single 2 Gy doses is not sufficient to destroy completely the MCTS as it does only affect the proliferating tumor cells in the surface layer. 

### 3.6. Irradiated MCTS Incubated with Au-Fe_3_O_4_ NHDs

**Figure 8:**
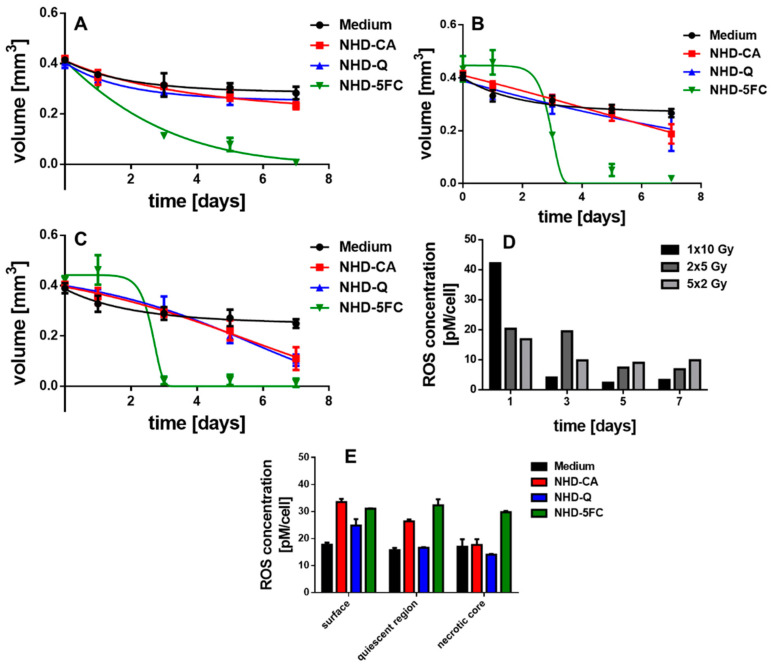
Temporal volume evolution of MCF-7 MCTS incubated in medium without (black dots) or with NHD-CA (red squares), NHD-Q (blue up-pointing triangles), and NHD-5FC (green down-pointing triangles) and irradiated with a single dose of 10 Gy (**A**), 2 single 5 Gy (**B**) and 5 single 2 Gy doses (**C**); intracellular reactive oxygen species (ROS) concentration of NHD-CA treated MCTS after X-ray irradiation (**D**), ROS concentration of the different MCTS regions after irradiation with a single 10 Gy dose (**E**).

**Figure 9:**
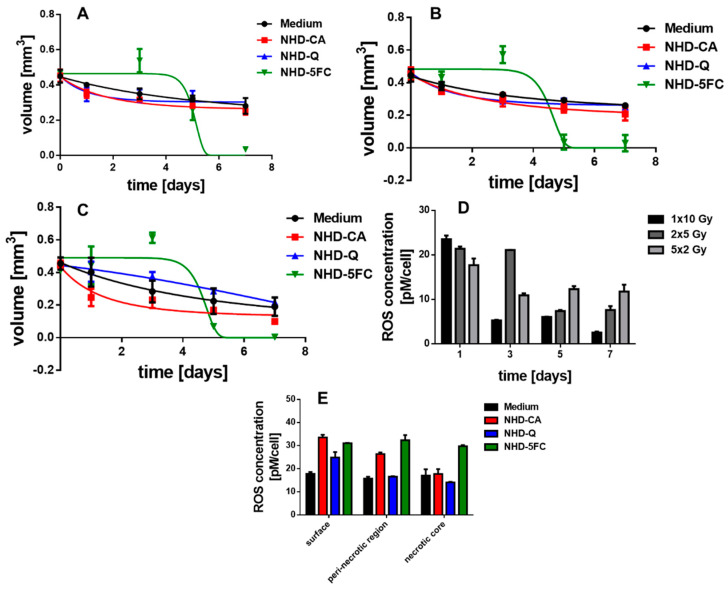
Temporal volume evolution of MDA-MB-231 MCTS incubated in medium without (black dots) or with NHD-CA (red squares), NHD-Q (blue up-pointing triangles), and NHD-5FC (green down-pointing triangles) and irradiated with one single dose of 10 Gy (**A**), 2 single 5 Gy doses (**B**) and 5 single 2 Gy doses (**C**); intracellular ROS concentration of NHD-CA treated MCTS after irradiation (**D**), ROS concentration of the different MCTS regions after irradiation with a single 10 Gy dose (**E**).

**Figure 10:**
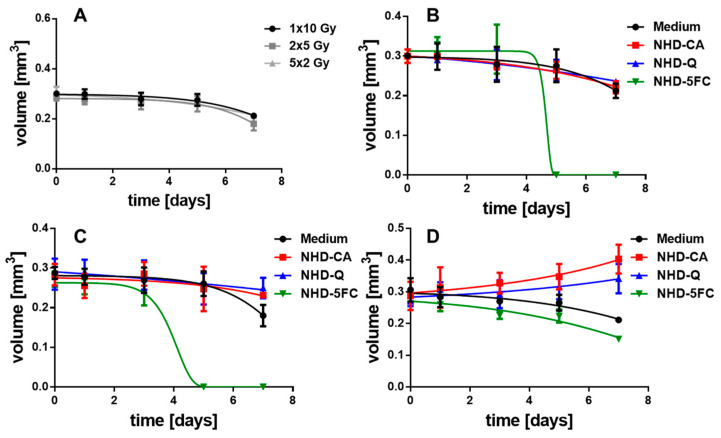
Temporal volume evolution of MCF-10A MCS incubated either in cell culture medium without (**A**) or over 72 h with NHD-CA, NHD-Q, and NHD-5FC and exposed to one single 10 Gy dose (**B**), 2 single 5 Gy doses (**C**) or 5 single 2 Gy doses (**D**).

The X-ray dose enhancing performance of the NHD-CA, NHD-Q, and NHD-5FC in MCF-7 and MDA-MB-231 MCTS and the X-ray protection by these NHDs in MCF-10A MCS were studied by incubating the MCTS with NHD-CA, NHD-Q, or NHD-5FC for 72 h. Afterwards the NHD-loaded MCTS were irradiated with either a single dose of 10 Gy, 2 single 5 Gy doses or 5 single 2 Gy. The treatment of MCF-7, MDA-MB-231, and MCF-10A MCTS loaded with NHD-CA and NHD-Q with a single dose of 10 Gy resulted in slightly reduced volumes compared with those of the corresponding MCTS grown in NHD-free medium Figure 8A, Figure 9A and Figure 10B, Figures S12, S16 and S20: 1rst and 2nd row, respectively). In contrast, intracellular NHD-5FC were found to enhance the impact of X-rays on the MCF-7 and MDA-MB-231 MCTS by drastically reducing their volumes due to visibly eradicating tumor cells after 4 days of growth (Figure 8A, Figure 9A and Figure 10B, Figures S12, S16 and S20: 3rd row). 

However, the NHD-CA and NHD-Q exhibited a considerably larger X-ray irradiation-enhancing effect on MCF-7 MCTS when irradiated with 2 single doses of 5 Gy and 5 single doses of 2 Gy (Figure 8B,C, Figures S13 and S14: 1rst and 2nd row), where the NHD-CA are more efficient than the NHD-Q. In the case of the MDA-MB-231 MCTS, the X-ray dose-enhancing effect of the NHD-CA was considerably smaller for the fractionated X-ray irradiation treatment, whereas the NHD-Q acted as radioprotector (Figure 9B,C, Figures S17 and S18: 1rst and 2nd row). When treated with fractionated X-ray irradiation the intracellular NHD-5FC caused the complete destruction of the breast tumor spheroids 3 days after X-ray irradiation (Figure 8B,C and Figure 9B,C, Figures S13, S14, S17 and S18: 3rd row).

### 3.7. Irradiated MCF-10 A MCS Incubated with Au-Fe_3_O_4_ NHDs

In contrast, the MCF-10A MCS being incubated before with the NHD-CA and NHD-Q and subsequently exposed to fractionated X-radiation (2 single 5 Gy doses) did not decrease in volume (Figure 10C, Figure S21: 1rst and 2nd row) but increased in volume for fractionated X-radiation with 5 single 2 Gy doses (Figure 10D, Figure S22: 1st and 2nd row). In comparison, the MCS cultivated in medium without NHDs und exposed to a single 10 Gy dose, 2 single 5 Gy doses, or 5 single 2 Gy doses decreased in volume over 7 days (Figure 10A). These results unambiguously demonstrate that the NHD-CA and NHD-Q act as radioprotectors in healthy epithelial cells. CA and Q are redox active and efficiently quench ROS in healthy cells containing ROS at low concentrations. Moreover, for the fractionated X-radiation treatment with 5 single 2 Gy doses intracellular NHD-5FC did not completely eradicate the MCF-10A MCS but only slightly reduce the MCS volume (Figure 10D, Figure S22: 3rd row), whereas the NHD-5FC completely destroyed the MCS when exposed to a single 10 Gy dose or 2 single 5 Gy doses (Figure 10B,C, Figures S20 and S21: 3rd row). 

### 3.8. ROS Generation in Irradiated MCTS

The growth curves of MCF-7 and MDA-MB-231 MCTS in Figure 8 and Figure 9 suggest that interactions between X-rays and the NHDs in MCTS affect intracellular ROS concentrations. For this reason, the ROS concentration of X-ray irradiated MCF-7 and MDA-MB-231 MCTS incubated in medium without and with NHD-CA, NHD-Q, or NHD-5FC was measured (Figure 8D,E and Figure 9D,E, Figures S15 and S19). One day after X-radiation treatment with a single 10 Gy dose the ROS concentration in MCF-7 MCTS with internalized NHD-CA reached a value of 43 pM/cell, whereas treatment with 2 single 5 Gy doses and 5 single 2 Gy doses led to ROS concentration values of 20 pM/cell and 17 pM/cell, respectively (Figure 8D). These ROS concentration values decreased in a different manner. The ROS value for the single 10 Gy dose dropped to 5 pM/cell after 7 days, whereas the ROS value for the 5 single 2 Gy doses fell off to 10 pM/cell. The same experiments were performed on NHD-CA loaded MBA-MB-231 MCTS: the value of the ROS concentration 1 day after irradiation with a single 10 Gy dose is 22 pM/cell and decayed to 3 pM/cell after 7 days. In comparison, the ROS concentration for the 5 single 2 Gy doses was 17 pM/cell at day 1 and decreased to 12 pM/cell.

On the other hand, the ROS concentration of NHD-Q- and NHD-5FC-loaded MCF-7 MCTS exposed to a single 10 Gy dose attained 40% smaller values, and the MCF-7 MCTS without NHDs yielded a 60% lower ROS concentration value (Figure S15). Compared with intracellular NHD-CA, MCF-7 containing NHD-Q and NHD-5FC and exposed to 2 single doses of 5 Gy and 5 single doses of 2 Gy, respectively, showed slightly smaller ROS concentration values. Similar results were obtained for NHD-free and NHD-Q loaded MDA-MB-231 MCTS when irradiated with a single 10 Gy dose, 2 single 5 Gy doses and 5 single 2 Gy doses (Figure S19). The NHD-5FC loaded MDA-MB-231 irradiated with equal X-radiation doses exhibited ca 30% larger ROS concentration values than these MCTS containing NHD-CA. 

Figure 8E and Figure 9E present the intracellular ROS concentration in the proliferating (surface), hypoxic (quiescent), and necrotic cells of MCF-7 and MDA-MB-231 MCTS incubated in medium containing NHD-CA, NHD-Q, and NHD-5FC, respectively, and then exposed to a single 10 Gy dose. NHD-CA in MCF-7 showed the best performance as ROS-enhancing agents: they increased the ROS level relative to the medium by 60%, 24%, and 40% in proliferating (surface), hypoxic (quiescent), and necrotic MCF-7 cells, respectively (Figure 8E). On the other hand, in MDA-MB-231 MCTS the NHD-5FC act as best ROS enhancing agents by raising the ROS level in the surface, quiescent, and necrotic region by 40%, 47%, and 44%, respectively, whereas the NHD-CA caused a higher ROS level increase only in the proliferating cells by 55%. 

We explain the main differences in the efficacy of the NHDs in the MCF-7 and MDA-MB-231 MCTS by different oxygen and ROS gradients (Figure 8E and Figure 9E). The interaction of X-rays with functionalized Au-Fe_3_O_4_ NHDs gave rise to the generation of O_2_^−^^•^ by electron emission from the Au component and simultaneously, provided the Fenton-catalyzed formation of ^•^OH radicals on the Fe_3_O_4_ surfaces. O_2_^−•^ generation requires oxygen, whereas ^•^OH emerges from H_2_O_2_. The intracellular ROS concentration was measured using 2′–7′dichlorofluorescin diacetate (DCFH-DA) which is mainly sensitive to H_2_O_2_. X-ray-induced activation of Au-Fe_3_O_4_ NHDs for ROS formation is directly coupled with X-ray-triggered release of the surface-bound agents Q, CA, and 5FC into the cytoplasm. The anti-metabolite drug 5FC destroyed tumor cells by inhibiting RNA and DNA synthesis and, thereupon, destroyed the cellular self-defense system. The flavonoids CA and Q acted in breast tumor cells as superoxide scavenger by converting O_2_^−^^•^ to H_2_O_2_, which increased the intracellular ROS concentration as being detected with the H_2_O_2_ sensitive DCF-DA assay. On the other hand, Q and CA operated as radioprotectors in the healthy MCF-10A cells whose antioxidant defense system counterbalanced the ROS. This enables Q and CA to scavenge ROS that are generated by X-ray irradiation. To summarize, NHD-CA in the MCF-7 and MDA-MB-231 MCTS showed the highest potential as X-ray dose enhancers and simultaneously impeded glycolytic metabolism by blocking MCT4 through X-ray-induced release of CA. On the other hand, NHD-CA in X-ray irradiated MCF-10 MCTS acted as the most capable radioprotectors.

## 4. Conclusions

Au-Fe_3_O_4_ nanoheterodimers (NHD) were functionalized with the natural and synthetic anticancer drugs caffeic acid (CA), quercetin (Q) and 5-fluorocytidine (5FC). Their X-ray irradiation dose-enhancing potential and chemotherapeutic efficacy for bimodal cancer therapy were investigated in multicellular tumor spheroids (MCTS). MCTS were grown from the breast tumor MCF-7 and MDA-MB-231cell lines, and for comparison from healthy breast epithelial MCF-10A cells. The MCF-7, MDA-MB-231MCTS and MCF-10A MCS were incubated with NHD-CA, NHD-Q, or NHD-5FC and then exposed to fractionated X-ray irradiation comprising either a single 10 Gy dose, 2 daily single 5 Gy doses or 5 daily single 2 Gy doses. The NHD-CA, NHD-Q, and NHD-5FC affected the growth of X-ray irradiated and non-irradiated MCTS in a different manner. The impact of the NHDs on the glycolytic metabolism due to oxygen deprivation inside MCTS was examined by measuring lactate secretion and glucose uptake of non-irradiated or X-ray irradiated MCTS. The NHDs were shown to act as X-ray irradiation dose enhancers in MCF-7 and MCF-10A MCTS, but the NHD-CA and NHD-Q served as radioprotector in MCF-10A MCS. These results were explained with the differences in the ROS concentration and antioxidant defense system of tumor cells in comparison with healthy cells. The X-ray dose enhancing performance of the NHDs consists in the generation of the reactive oxygen species O2^−^^•^ and ^•^OH. Simultaneously, the interaction between the X-rays and the NHDs causes the release of the drugs CA, Q, and 5FC into the cytosol. The redox-active CA and Q provide additional generation of H_2_O_2_ in the cancerous MCF-7 and MDA-MB-231 cells, whereas the antimetabolite 5FC exerts a greater cytotoxic effect in MCF-7 and MDA-MB-231 MCTS than in MCF-10A MCS. Moreover, CA and Q inhibited lactate secretion in the breast tumor spheroids and thereupon disturbed glycolytic reprogramming of normoxic tumor cells. In comparison with the NHD-Q, for the MCF-7 and MDA-MB-231 MCTS, the NHD-CA showed the highest potential as X-ray dose enhancing agents and simultaneously impede the glycolytic metabolism by inhibiting the MCT4. In a nutshell, the NHD-CA exhibit encouraging potential for application as nanotherapeutics in combined radiotherapy and chemotherapy, as they allow for radiation damage to hypoxic cells due to their bimodal action.

## Supplementary Materials

The following are available online at https://www.mdpi.com/article/10.3390/nano11051167/s1, Figure S1: UV-Vis absorption spectra of the NHD-CA (black line), NHD-Q (red line) and NHD-5FC (blue line) dispersed at a concentration of 40 μg/mL, Figure S2: Microscope images of the MCF-7 MCTS incubated with NHD-CA (first row), NHD-Q (second row) and NHD-5FC (third row) without irradiation, Figure S3: Cell viability of the MCF-7 MCTS after incubating for 72 h in NHD-free medium and with NHD-CA, NHD-Q and NHD-5FC; separated into the different spheroid regions, Figure S4: Microscope images of the MDA-MB-231 MCTS incubated with NHD-CA (first row), NHD-Q (second row) and NHD-5FC (third row) without irradiation, Figure S5: Cell viability of the MDA-MB-231 MCTS after incubating for 72 h without and with the various NHDs; separated into the different spheroid regions, Figure S6: Microscope images of the MCF-10 A MCS incubated with NHD-CA (first row), NHD-Q (second row) and NHD-5FC (third row) without irradiation, Figure S7: Cell viability of the MCF-10 A MCS after incubating for 72 h without and with the various NHDs; separated into the different spheroid regions, Figure S8: Lactate secretion (A) and glucose uptake (B) of the MCF-10 A MCS, Figure S9: Intracellular lactate concentration of the different regions of the MCTS loaded with NHD-CA and NHD-Q; MCF-7 MCTS (A), MDA-MB-231 MCTS (B), MCF-10 A MCS (C), Figure S10: Iron content of the separated MCF-7 MCTS regions cultivated in medium (A) or in medium with NHD-CA (B), NHD-Q (C) and NHD-5FC (D), Figure S11: Iron content of the separated MCF-10 A MCS regions cultivated in medium (A) or in medium with NHD-CA (B), NHD-Q (C) and NHD-5FC (D), Figure S12: Microscope images of the MCF-7 MCTS treated with NHD-CA (first row), NHD-Q (second row) and NHD-5FC (third row) irradiated with a single dose of 10 Gy at day 1; Figure S13: Microscope images of the MCF-7 MCTS treated with NHD-CA (first row), NHD-Q (second row) and NHD-5FC (third row) irradiated with 2 single 5 Gy doses; Figure S14: Microscope images of the MCF-7 MCTS treated with NHD-CA (first row), NHD-Q (second row) and NHD-5FC (third row) irradiated with 5 single 2 Gy doses, Figure S15: Intracellular ROS concentration in MCF-7 MCTS in medium (A) of loaded with NHD-Q (B) or NHD-5FC after irradiation with a single dose of 10 Gy, 2 single 5 Gy doses, or 5 single 2 Gy doses, Figure S16: Microscope images of the MDA-MB-231 MCTS treated with NHD-CA (first row), NHD-Q (second row) and NHD-5FC (third row) irradiated with a single dose of 10 Gy at day 1; Figure S17: Microscope images of the MDA-MB-231 MCTS treated with NHD-CA (first row), NHD-Q (second row) and NHD-5FC (third row) irradiated with 2 single 5 Gy doses from day 1 to 2; Figure S18: Microscope images of the MDA-MB-231 MCTS treated with NHD-CA (first row), NHD-Q (second row) and NHD-5FC (third row) irradiated with 5 single 2 Gy doses from day 1 to 5, Figure S19: Intracellular ROS concentration in MDA-MB-231 MCTS in medium (A) of loaded with NHD-Q (B) or NHD-5FC after irradiation with a single dose of 10 Gy, 2 single 5 Gy doses, or 5 single 2 Gy doses, Figure S20: Microscope images of the MCF-10 A MCS treated with NHD-CA (first row), NHD-Q (second row) and NHD-5FC (third row) irradiated with a single dose of 10 Gy at day 1; Figure S21: Microscope images of the MCF-10 A MCS treated with NHD-CA (first row), NHD-Q (second row) and NHD-5FC (third row) irradiated with 2 single 5 Gy doses from day 1 to 2; Figure S22: Microscope images of the MCF-10 A MCS treated with NHD-CA (first row), NHD-Q (second row) and NHD-5FC (third row) irradiated with 5 single 2 Gy doses from day 1 to 5.
