# Supplementary material for: Caffeic Acid, Quercetin and 5-Fluorocytidine-Functionalized Au-Fe3O4 Nanoheterodimers for X-ray-Triggered Drug Delivery in Breast Tumor Spheroids
Source: Nanomaterials (Basel). 2021 Apr 29;11(5):1167. doi: 10.3390/nano11051167 (PMC8146450; doi:10.3390/nano11051167)
Supplement: Supplementary file 1 [file nanomaterials-11-01167-s001.zip › nanomaterials-1159037-supplementary.pdf]

Supporting information

Caffeic acid, Quercetin and 5-Fluorocytidine functionalized Au-Fe_3O_4 Nanoheterodimers for X-Ray-Triggered drug delivery in breast tumor spheroids

Stefanie Klein [1]*, Luitpold V. R. Distel [2], Winfried Neuhuber [3] and Carola Kryschi [1]

[1] Department of Chemistry and Pharmacy, Physical Chemistry I and ICMM, Friedrich-Alexander University of Erlangen- Nuremberg, Egerlandstr. 3, D-91058 Erlangen, Germany; stefanie.klein@fau.de, carola.kryschi@fau.de
[2] Department of Radiation Oncology, Friedrich-Alexander University of Erlangen-Nuremberg, Universitätsstr. 27, D-91054 Erlangen Germany, luitpold.distel@fau.de
[3] Institute of Anatomy and Cell Biology, Chair of Anatomy I, Friedrich Alexander University Erlangen-Nuremberg, Krankenhausstr. 9, D-91054 Erlangen, Germany, winfried.neuhuber@fau.de
* Correspondence: stefanie.klein@fau.de

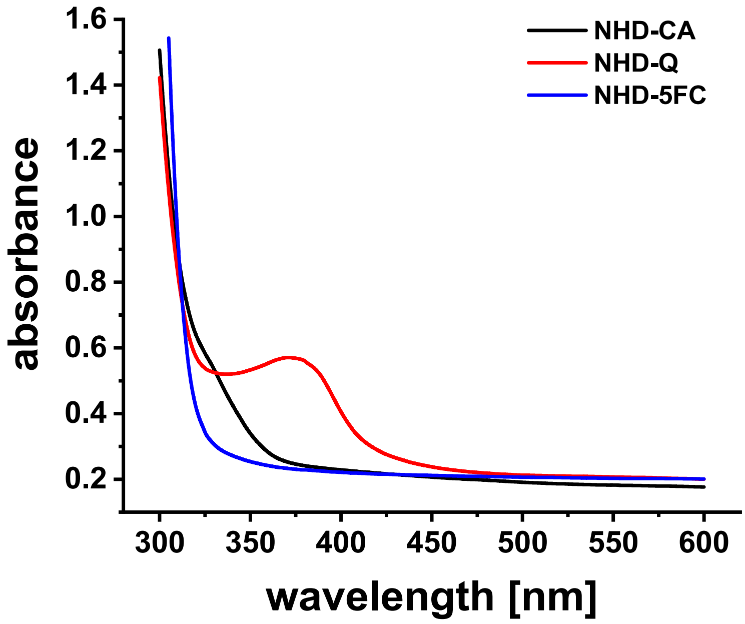

Figure S1: UV-Vis absorption spectra of the NHD-CA (black line), NHD-Q (red line) and NHD-5FC (blue line) dispersed at a concentration of 40 µg/mL.

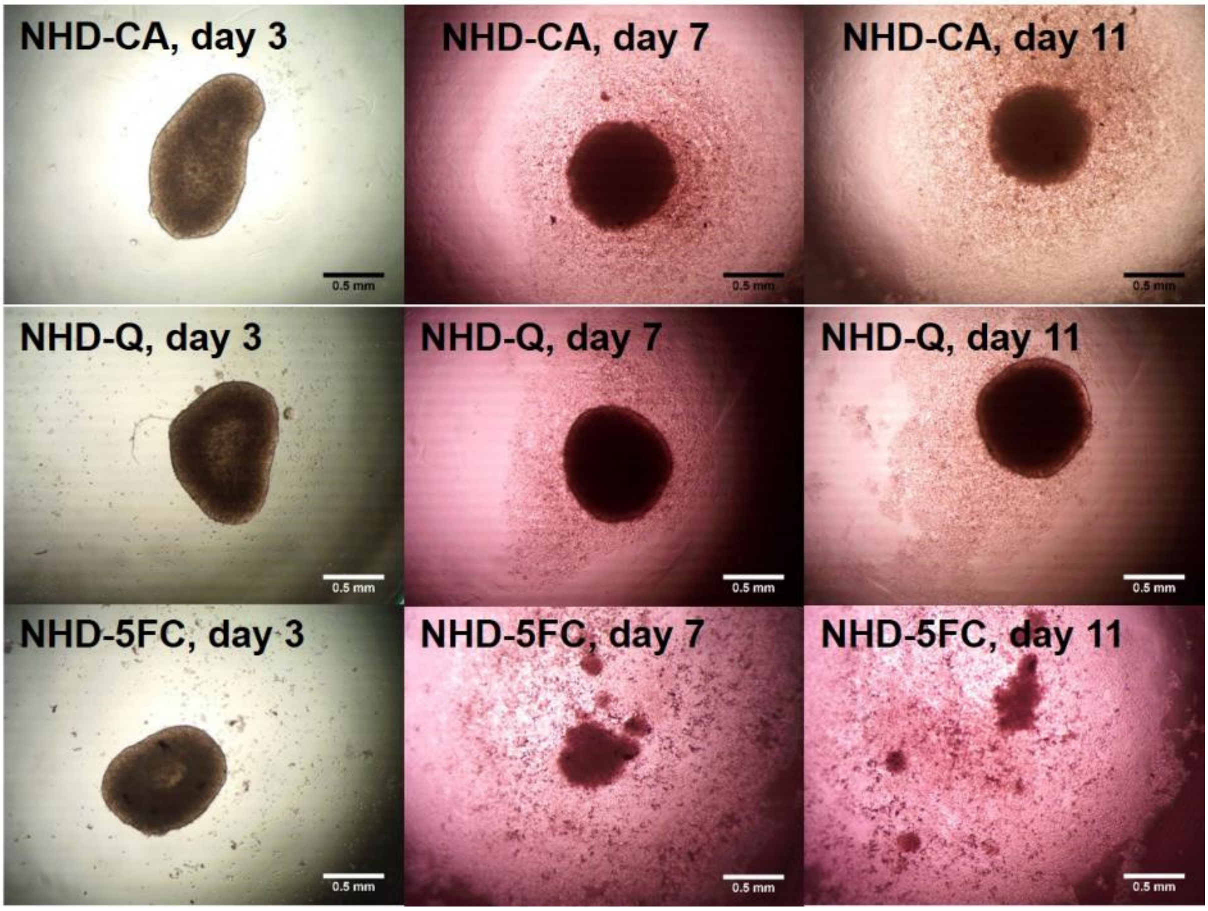

Figure S2: Microscope images of the MCF-7 MCTS incubated with NHD-CA (first row), NHD-Q (second row) and NHD-5FC (third row) without irradiation.

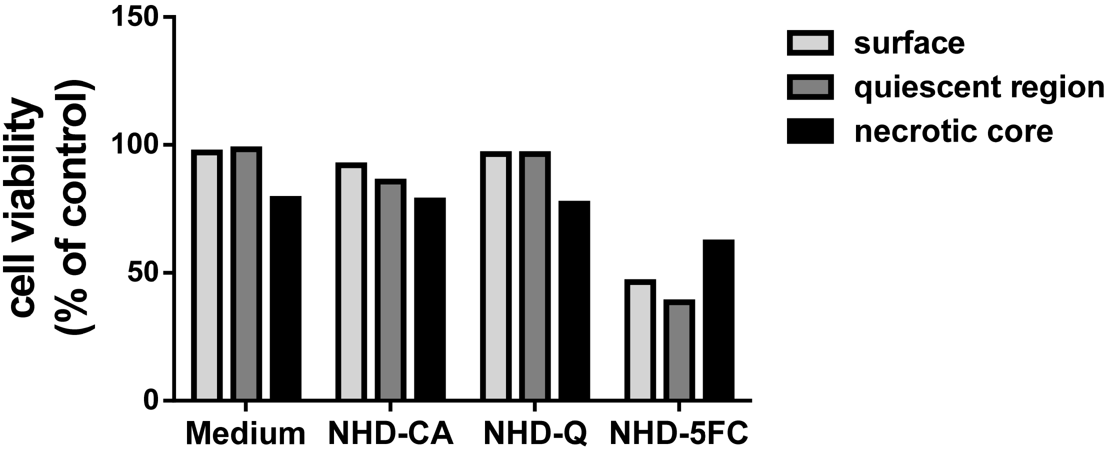

Figure S3: Cell viability of the MCF-7 MCTS after incubating for 72 h in NHD-free medium, and with NHD-CA, NHD-Q, and NHD-5FC; separated into the different spheroid regions.

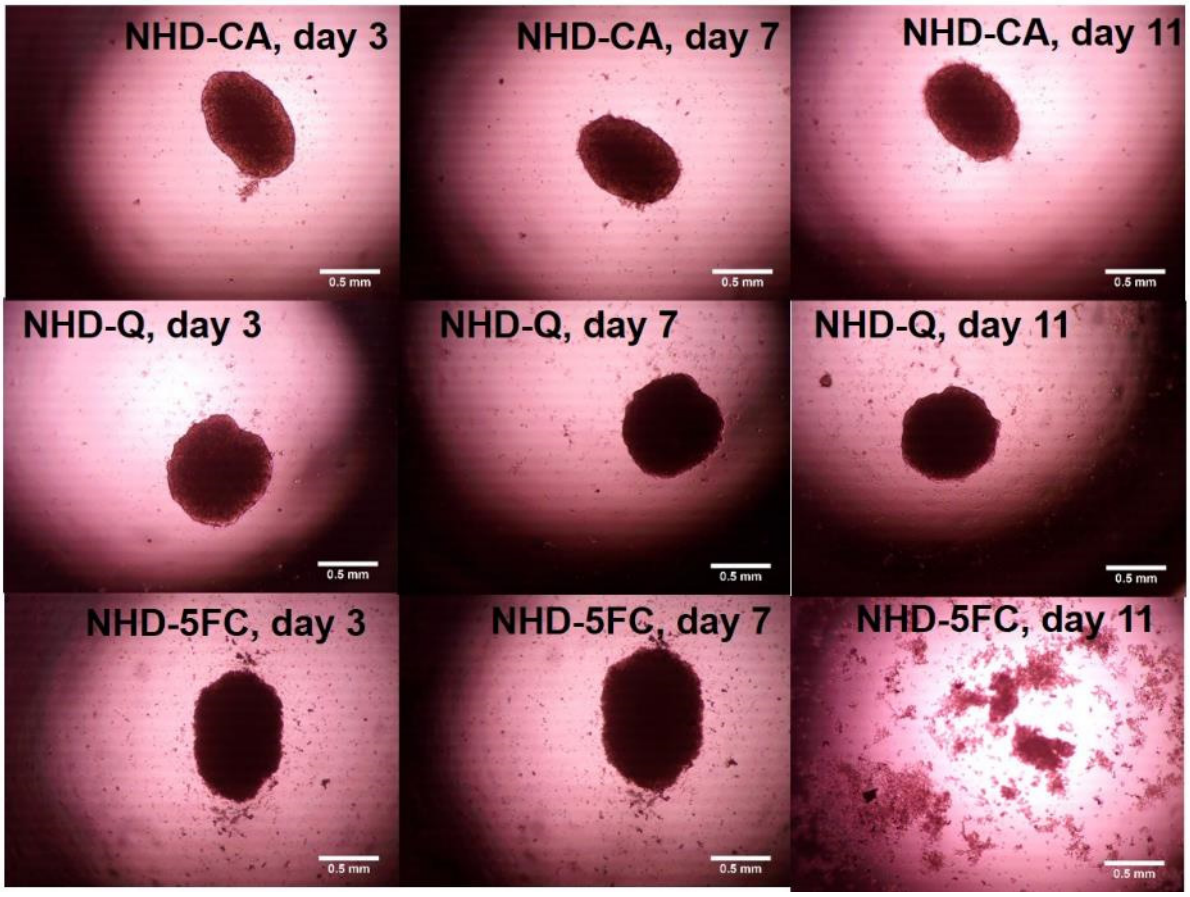

Figure S4: Microscope images of the MDA-MB-231 MCTS treated with NHD-CA (first row), NHD-Q (second row) and NHD-5FC (third row) without irradiation.

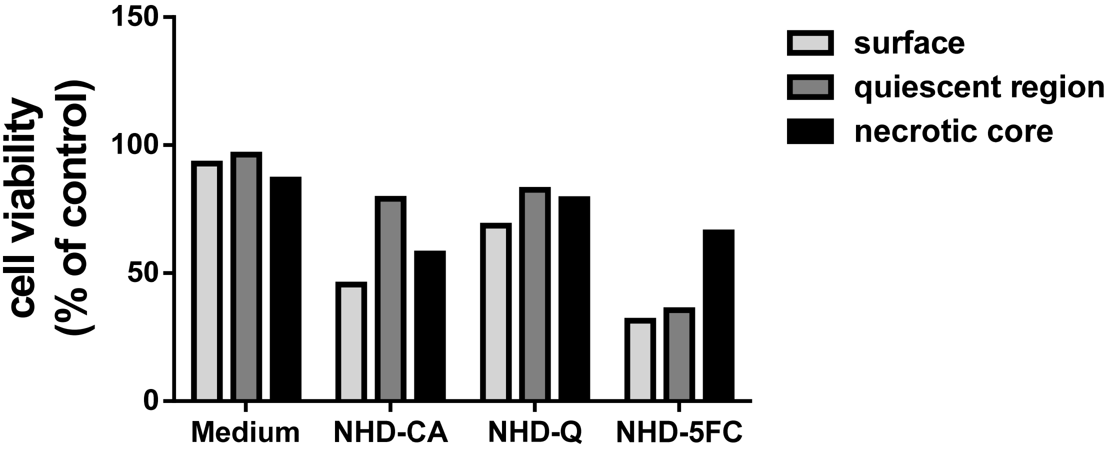

Figure S5: Cell viability of the MDA-MB-231 MCTS after incubation without and with the various NHDs for 72 h; separated into the different spheroid regions.

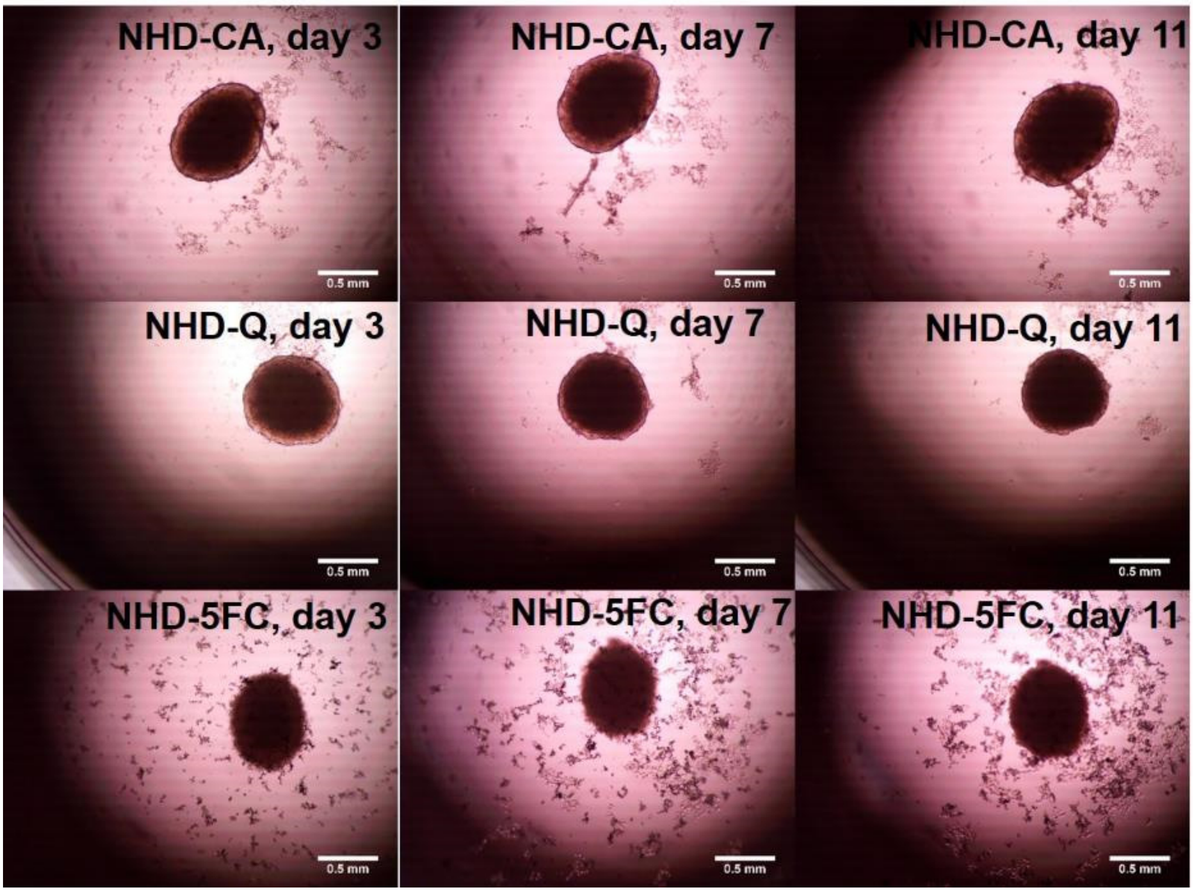

Figure S6: Microscope images of the MCF-10A MCS treated with NHD-CA (first row), NHD-Q (second row) and NHD-5FC (third row) without irradiation.

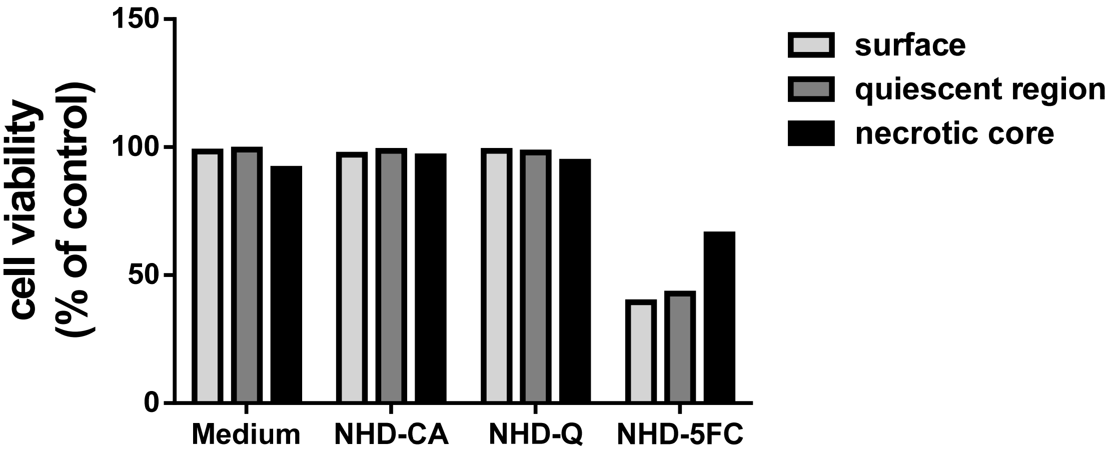

Figure S7: Cell viability of the MCF-10A MCS after incubation without and with the various NHDs for 72 h; separated into the different spheroid regions.

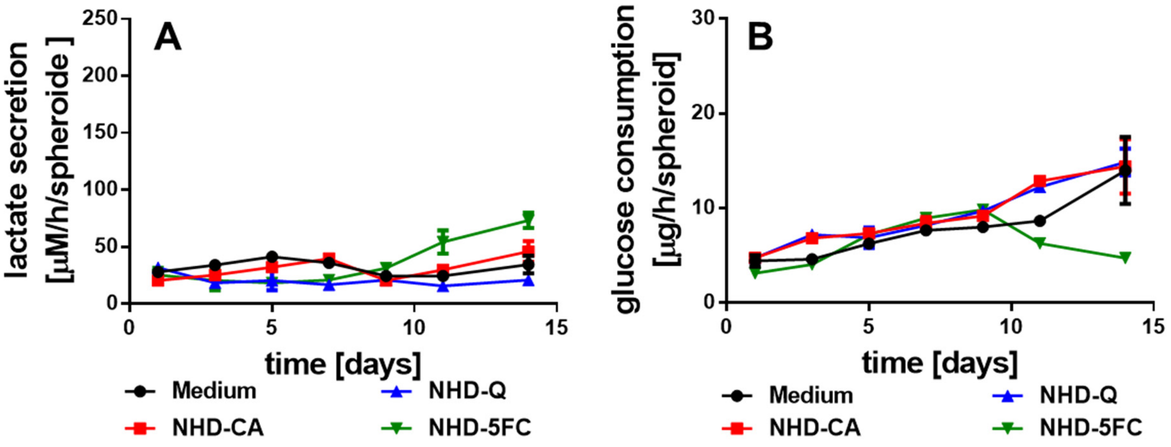

Figure S8: Lactate secretion (A) and glucose uptake (B) of the MCF-10 A MCS.

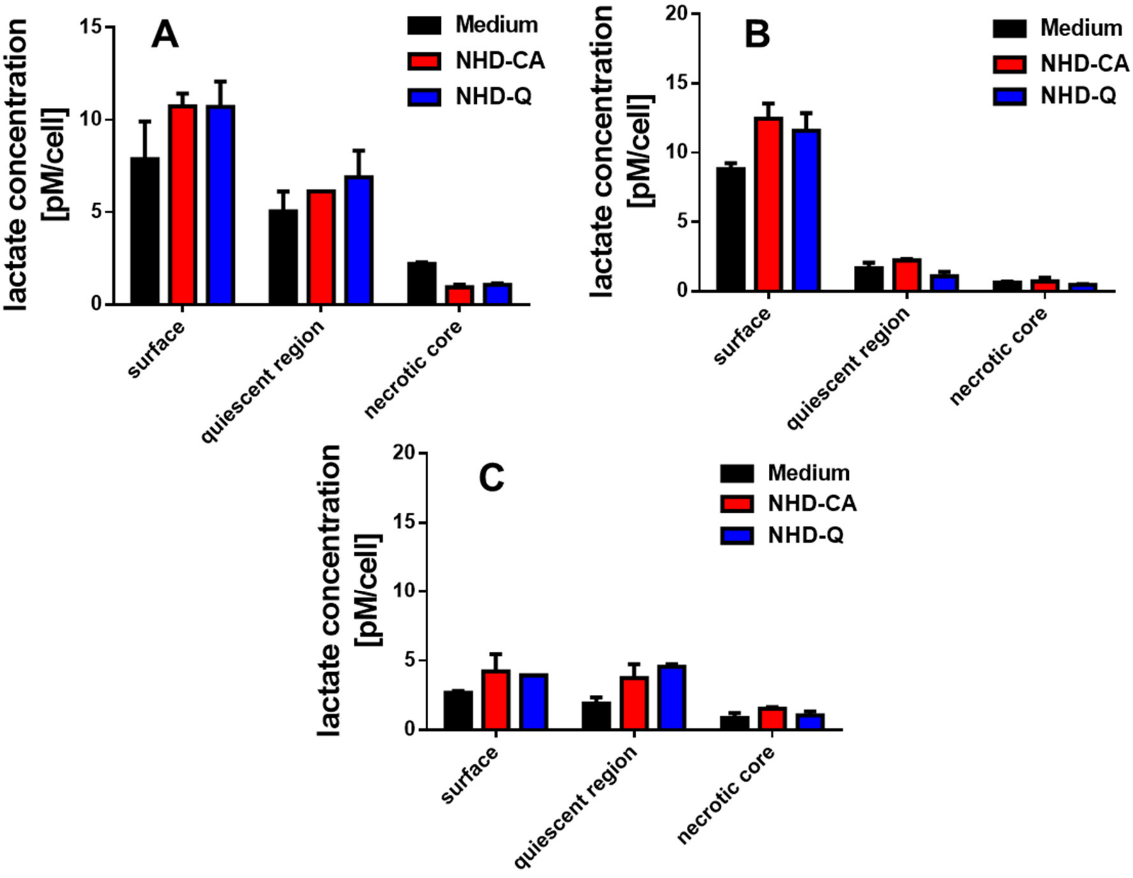

Figure S9: Intracellular lactate concentration of the different regions of the MCTS loaded with NHD-CA and NHD-Q; MCF-7 MCTS (A), MDA-MB-231 MCTS (B), MCF-10 A MCS (C).

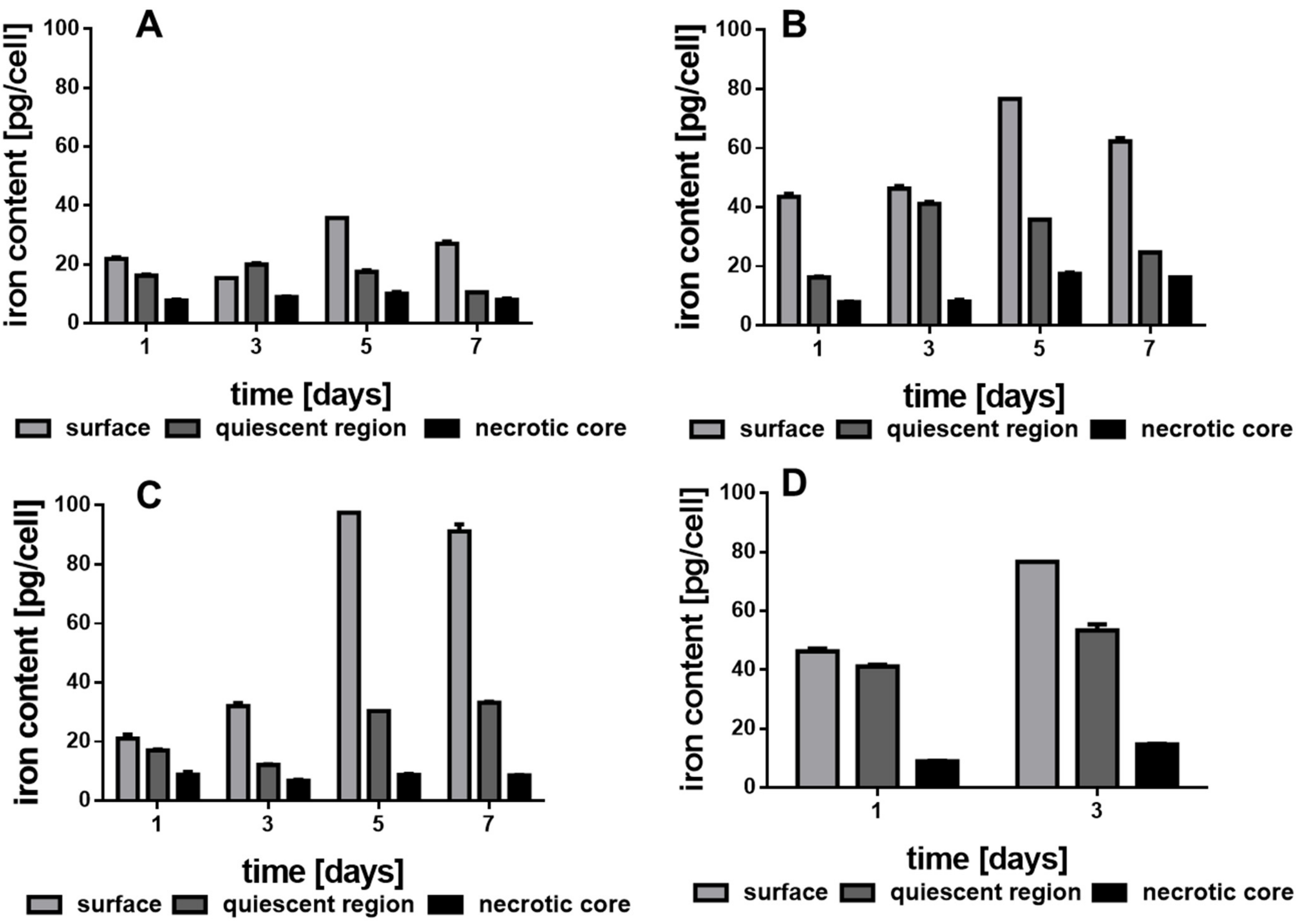

Figure S10: Iron content of the separated MCF-7 MCTS regions cultivated in medium (A) or in medium with NHD-CA (B), NHD-Q (C) and NHD-5FC (D).

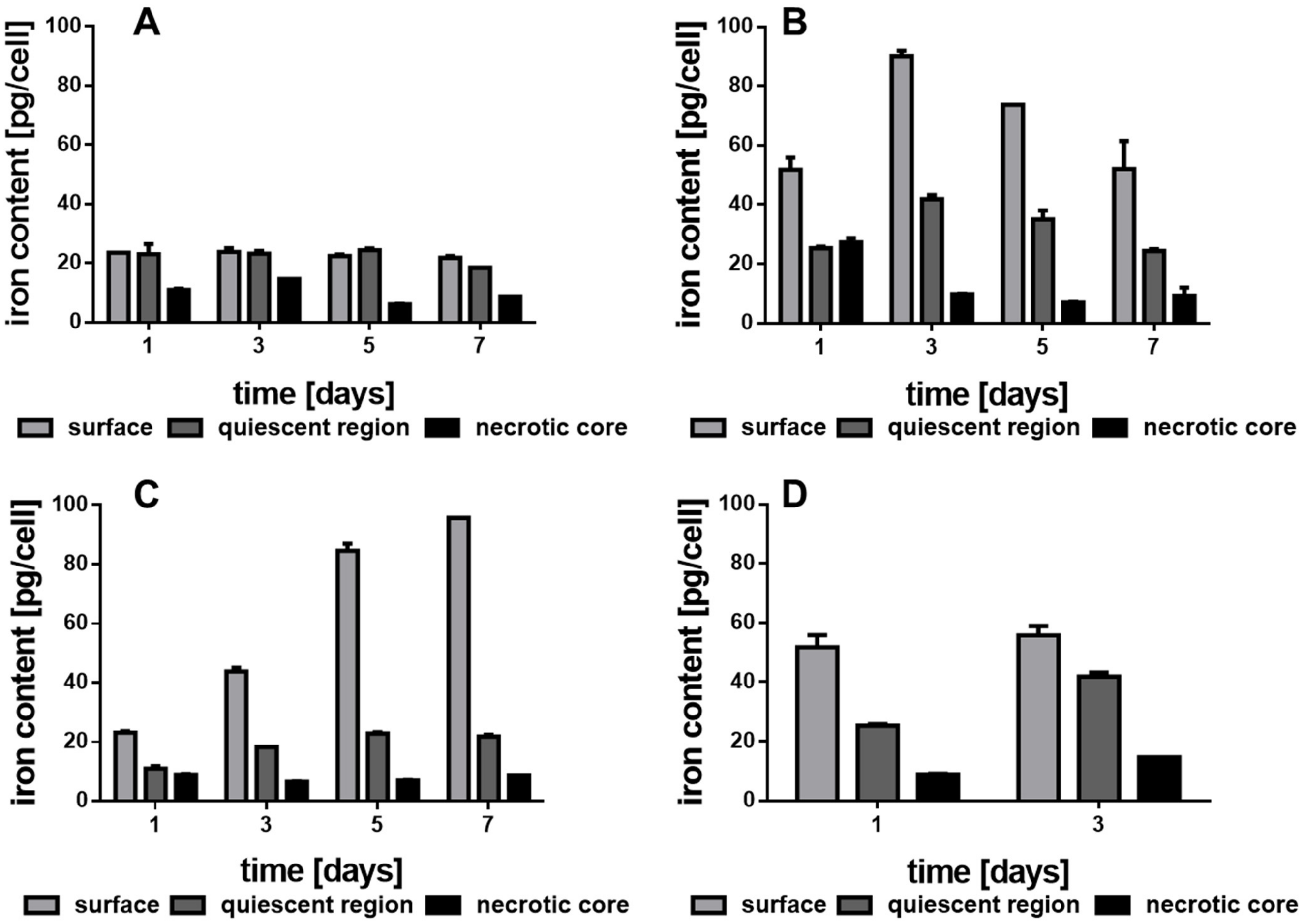

Figure S11: Iron content of the separated MCF-10 A MCS regions cultivated in medium (A) or in medium with NHD-CA (B), NHD-Q (C) and NHD-5FC (D).

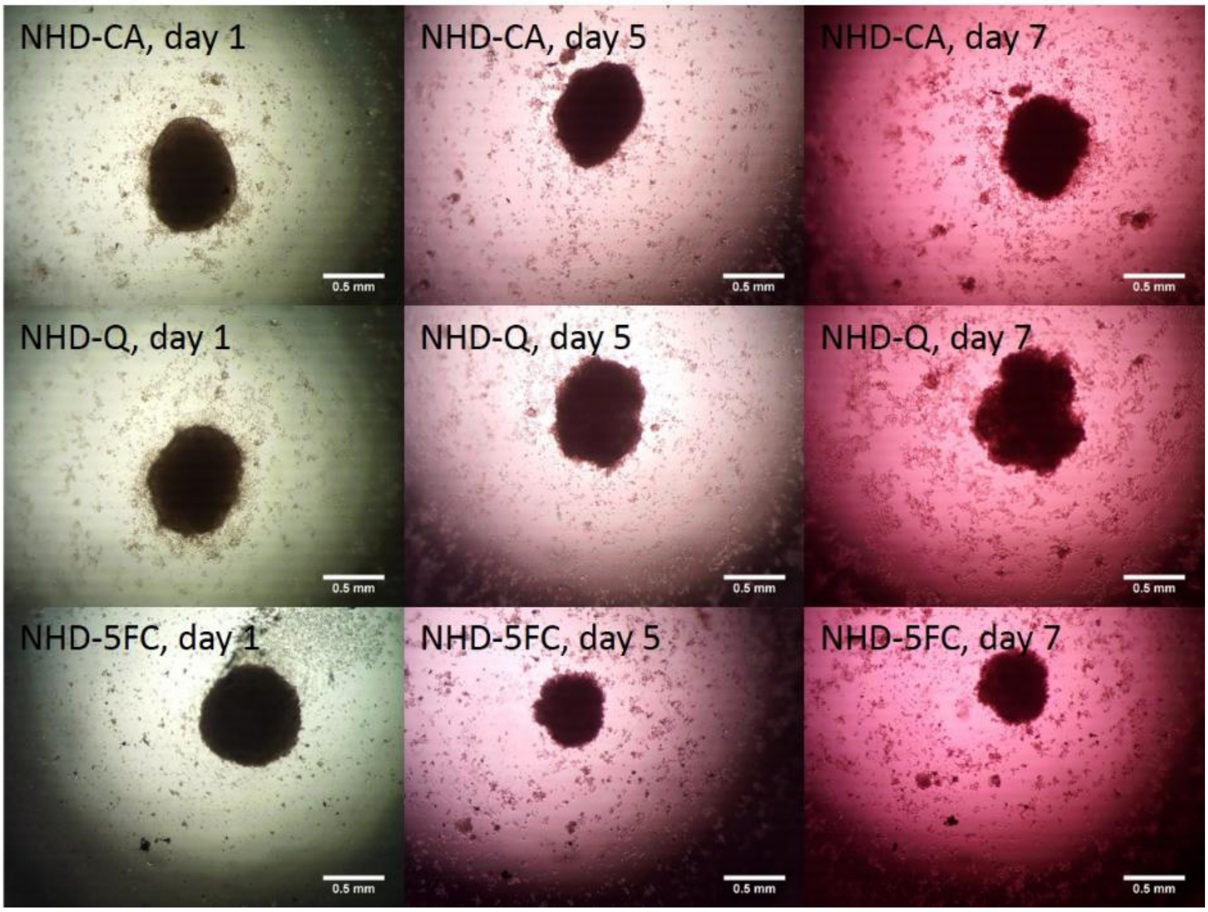

Figure S12: Microscope images of the MCF-7 MCTS treated with NHD-CA (first row), NHD-Q (second row) and NHD-5FC (third row) irradiated with a single dose of 10 Gy at day 1.

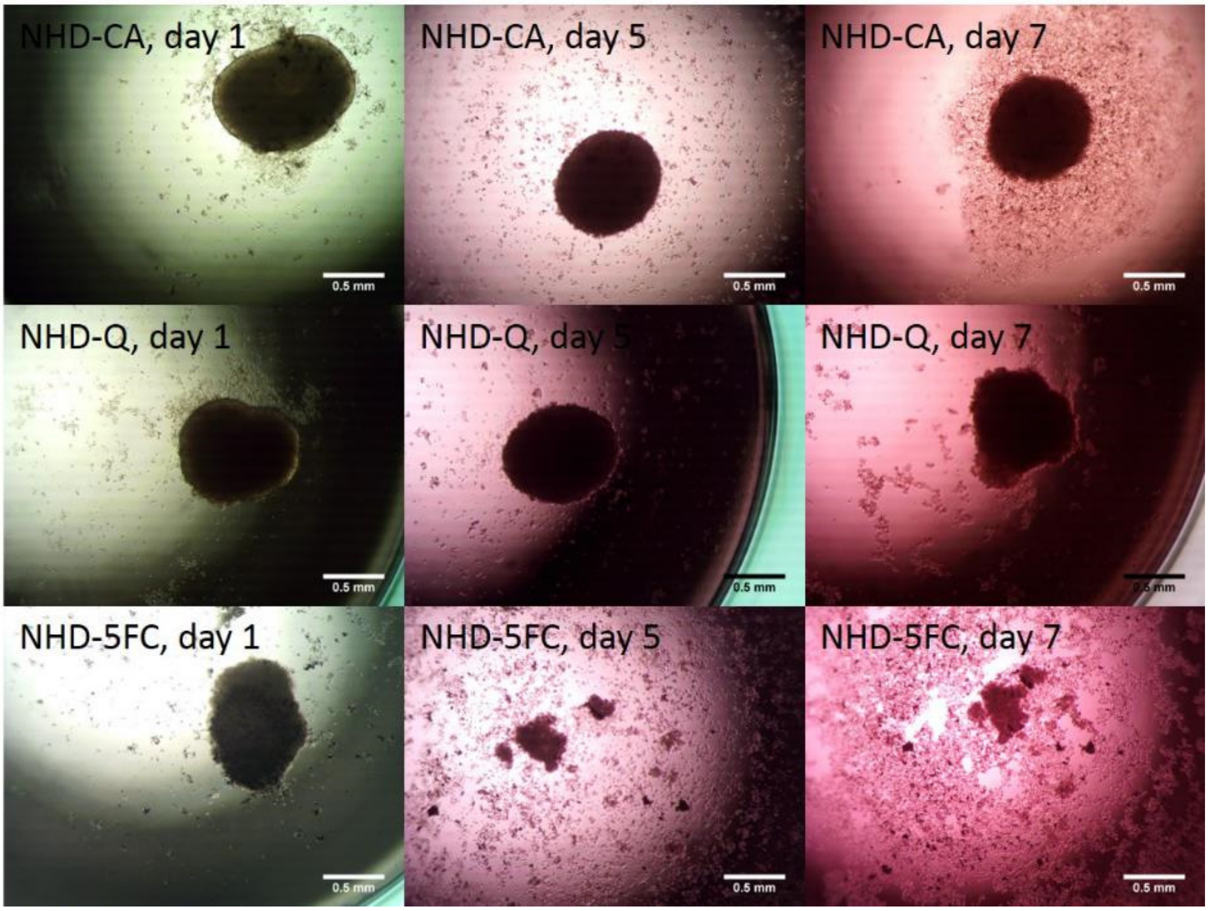

Figure S13: Microscope images of the MCF-7 MCTS treated with NHD-CA (first row), NHD-Q (second row) and NHD-5FC (third row) irradiated with 2 single 5 Gy doses.

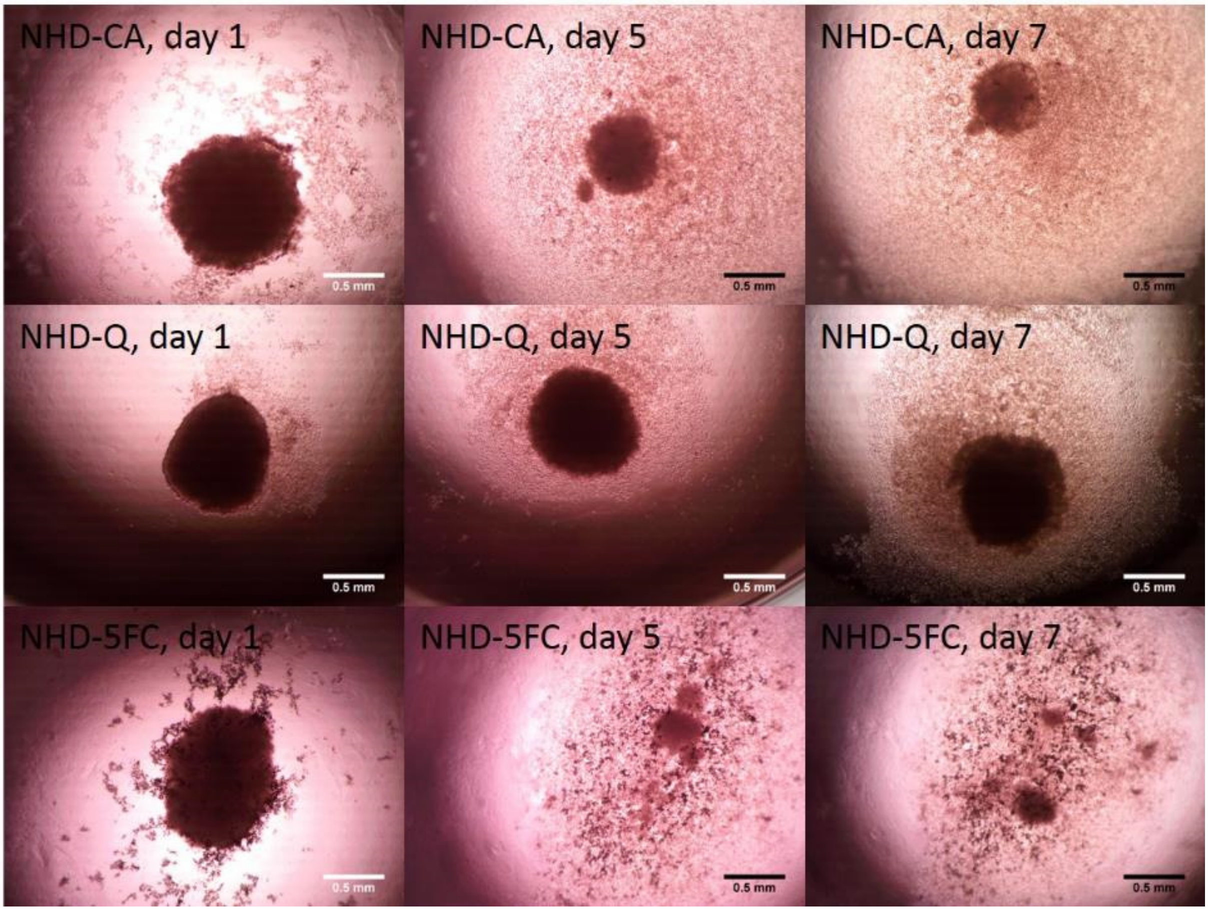

Figure S14: Microscope images of the MCF-7 MCTS treated with NHD-CA (first row), NHD-Q (second row) and NHD-5FC (third row) irradiated with 5 single 2 Gy doses.

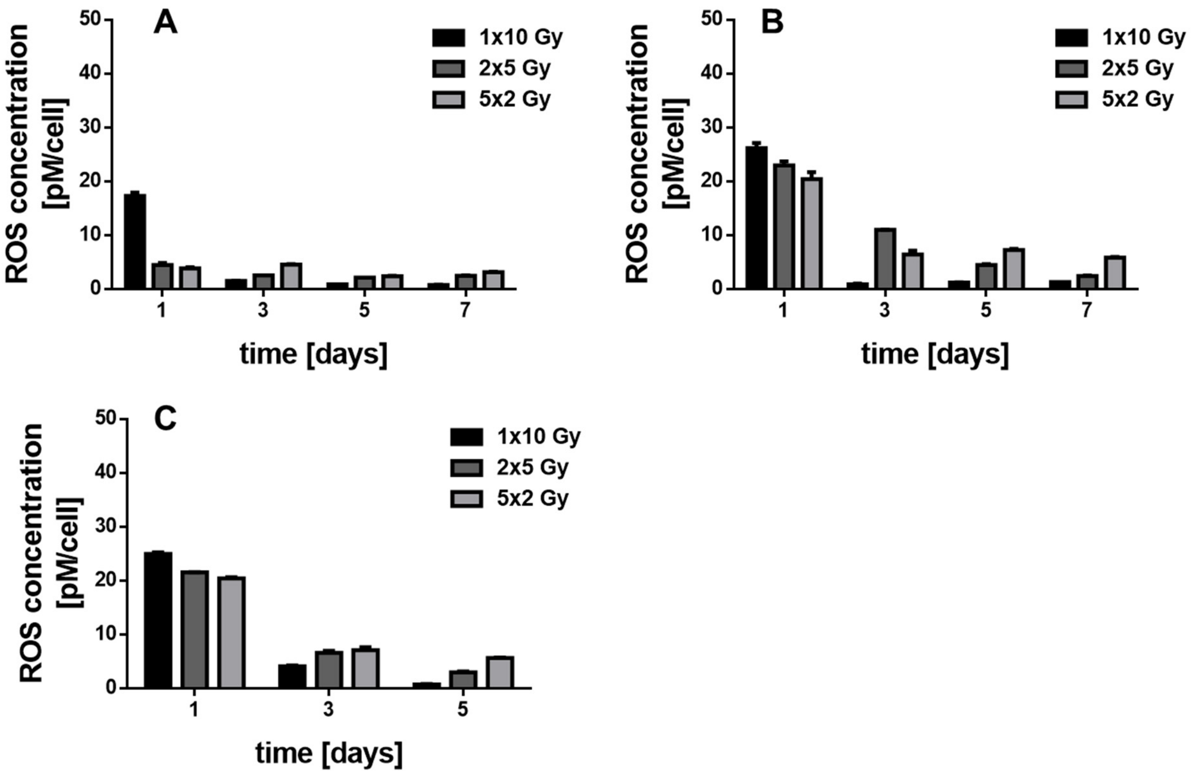

Figure S15: Intracellular ROS concentration in MCF-7 MCTS in medium (A) or loaded with NHD-Q (B) or NHD-5FC (C) after irradiation with a single dose of 10 Gy, 2 single 5 Gy doses, or 5 single 2 Gy doses.

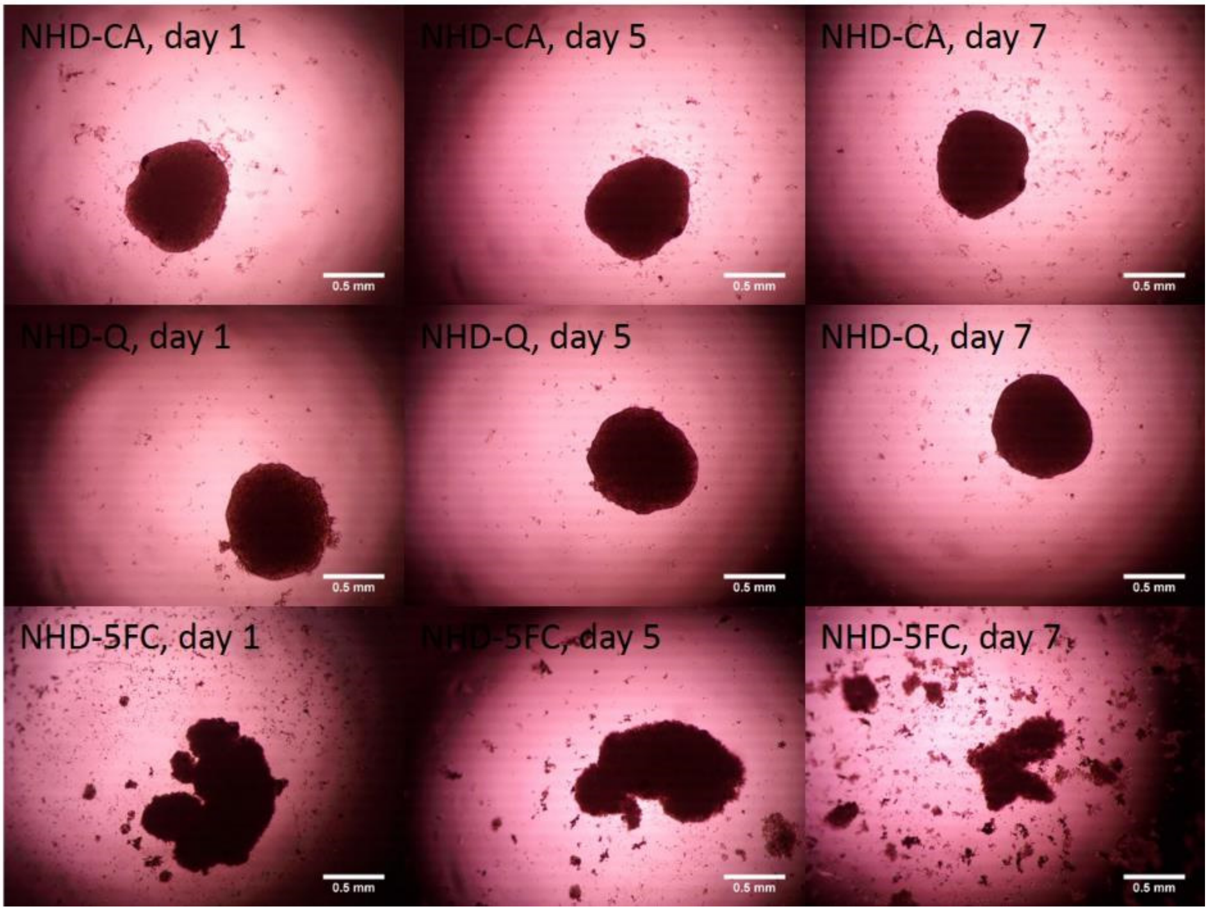

Figure S16: Microscope images of the MDA-MB-231 MCTS treated with NHD-CA (first row), NHD-Q (second row) and NHD-5FC (third row) irradiated with a single dose of 10 Gy at day 1.

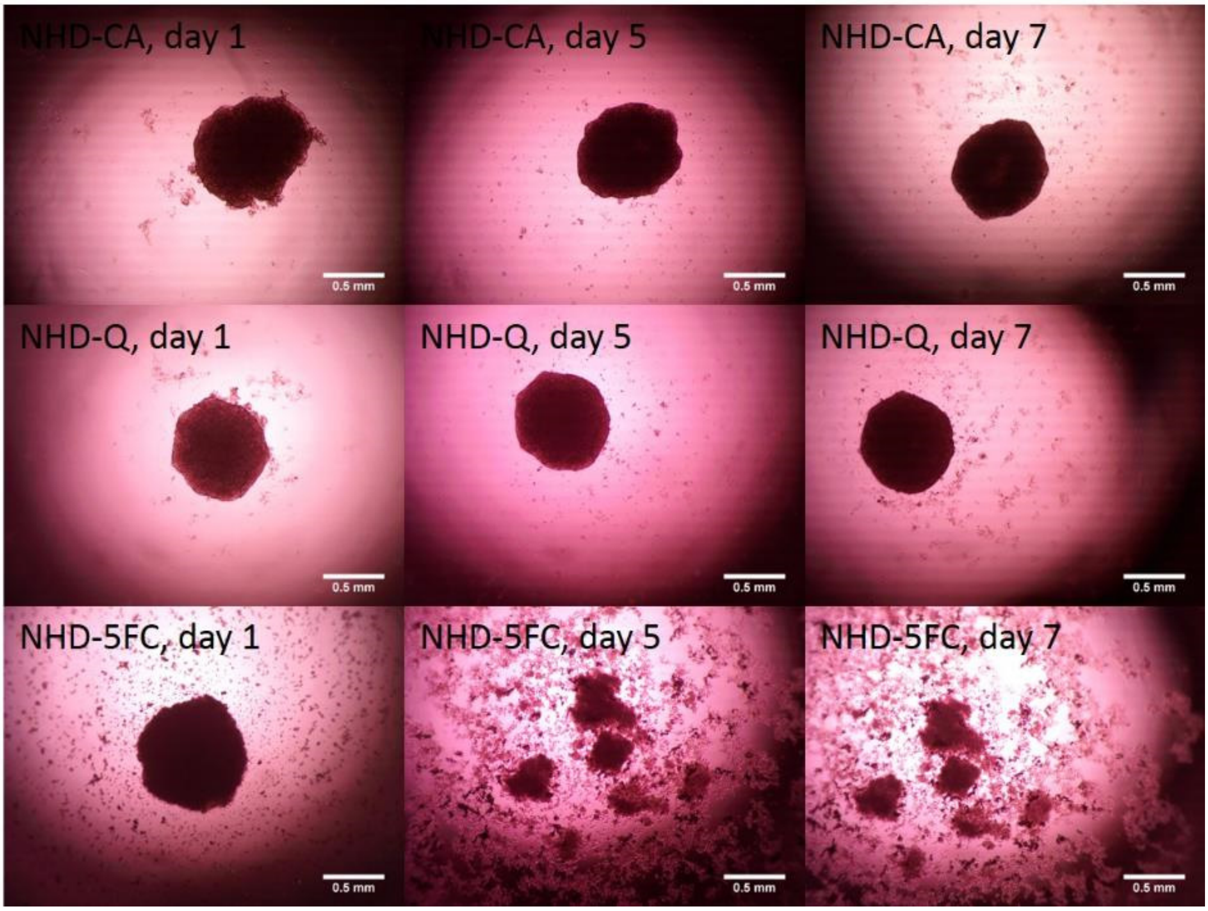

Figure S17: Microscope images of the MDA-MB-231 MCTS treated with NHD-CA (first row), NHD-Q (second row) and NHD-5FC (third row) irradiated with 2 single 5Gy doses from day 1 to 2.

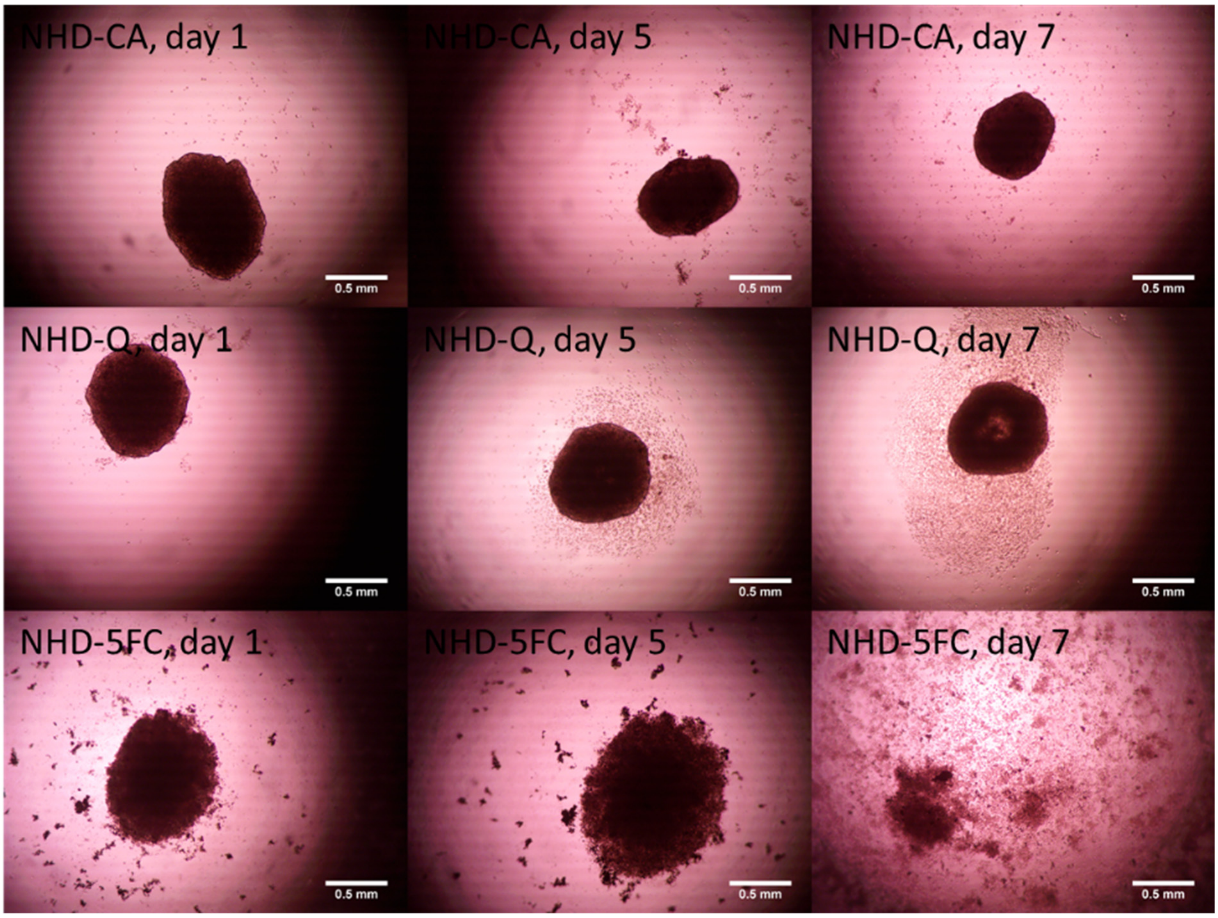

Figure S18: Microscope images of the MDA-MB-231 MCTS treated with NHD-CA (first row), NHD-Q (second row) and NHD-5FC (third row) irradiated with 5 single 2 Gy doses from day 1 to 5.

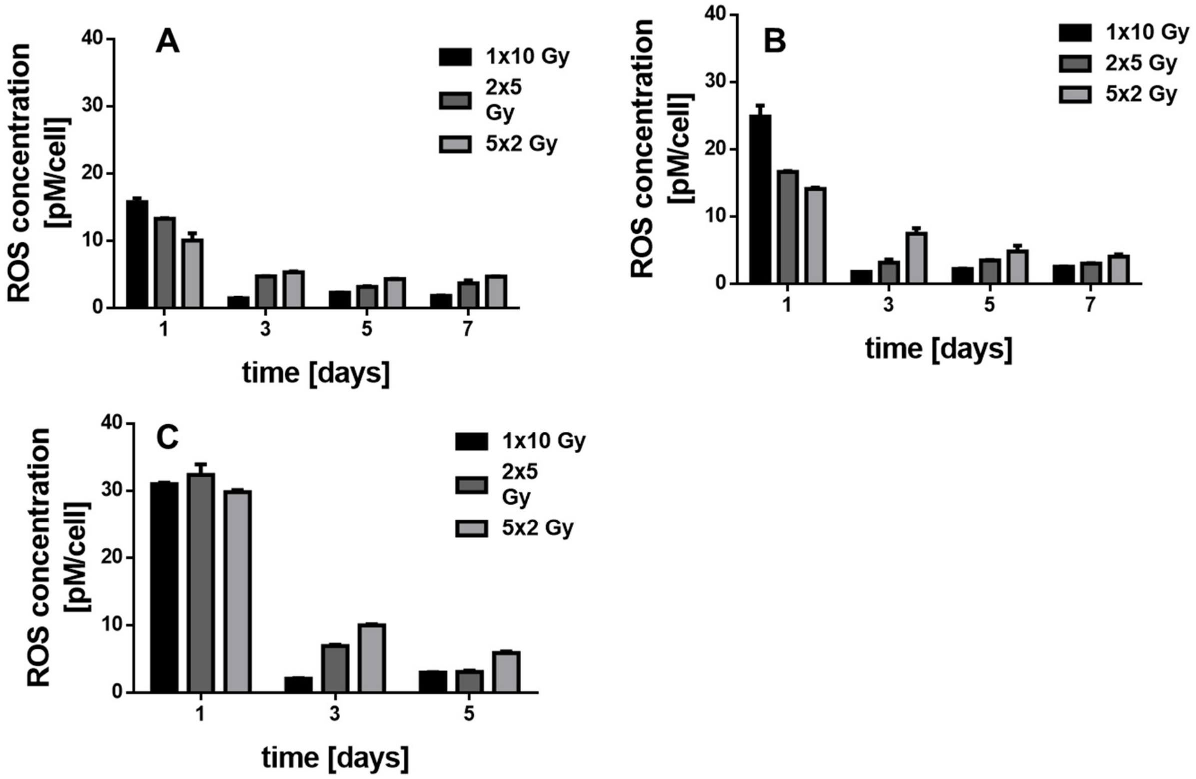

Figure S19: Intracellular ROS concentration in MDA-MD-231 MCTS in medium (A) or loaded with NHD-Q (B) or NHD-5FC (C) after irradiation with a single dose of 10 Gy, 2 single 5 Gy doses, or 5 single 2 Gy doses.

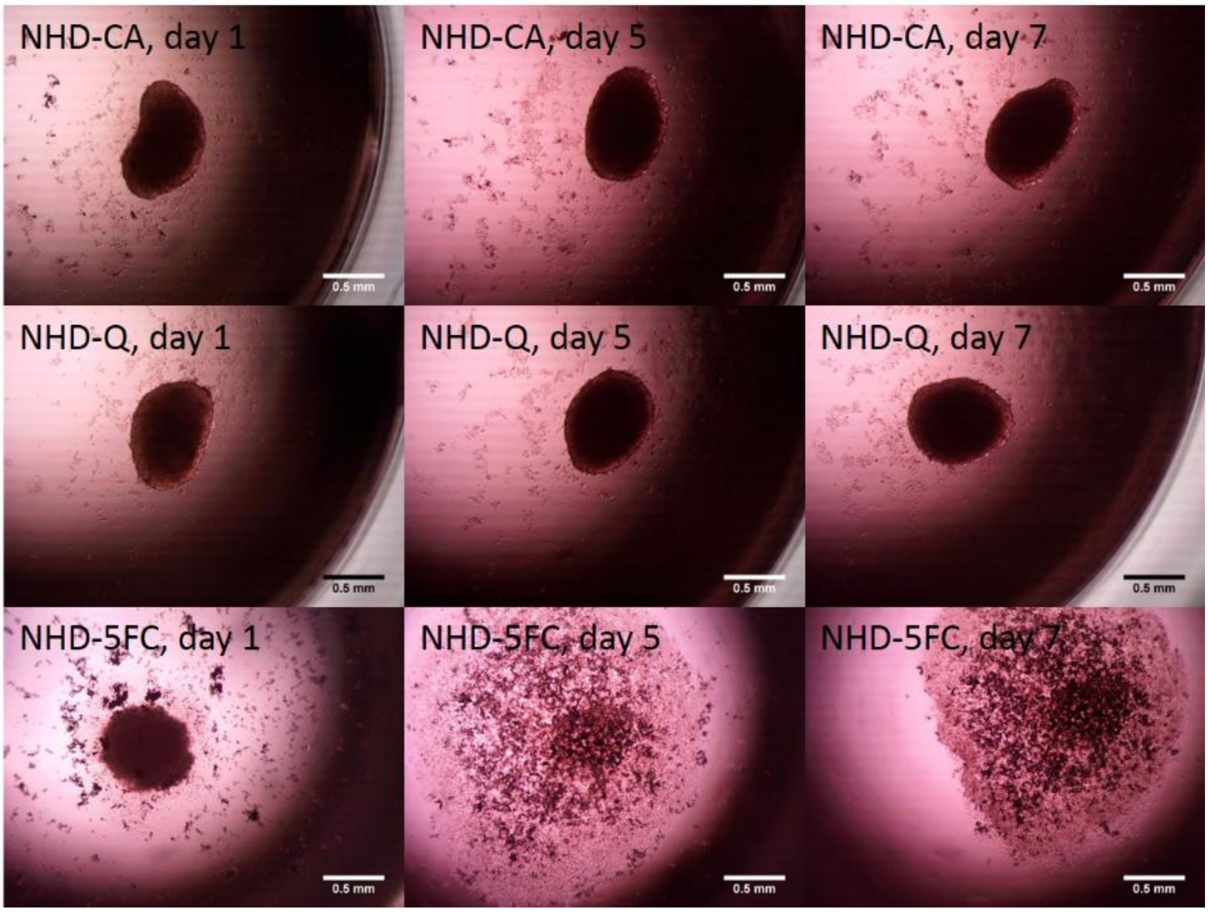

Figure S20: Microscope images of the MCF-10 A MCS treated with NHD-CA (first row), NHD-Q (second row) and NHD-5FC (third row) irradiated with a single dose of 10 Gy at day 1.

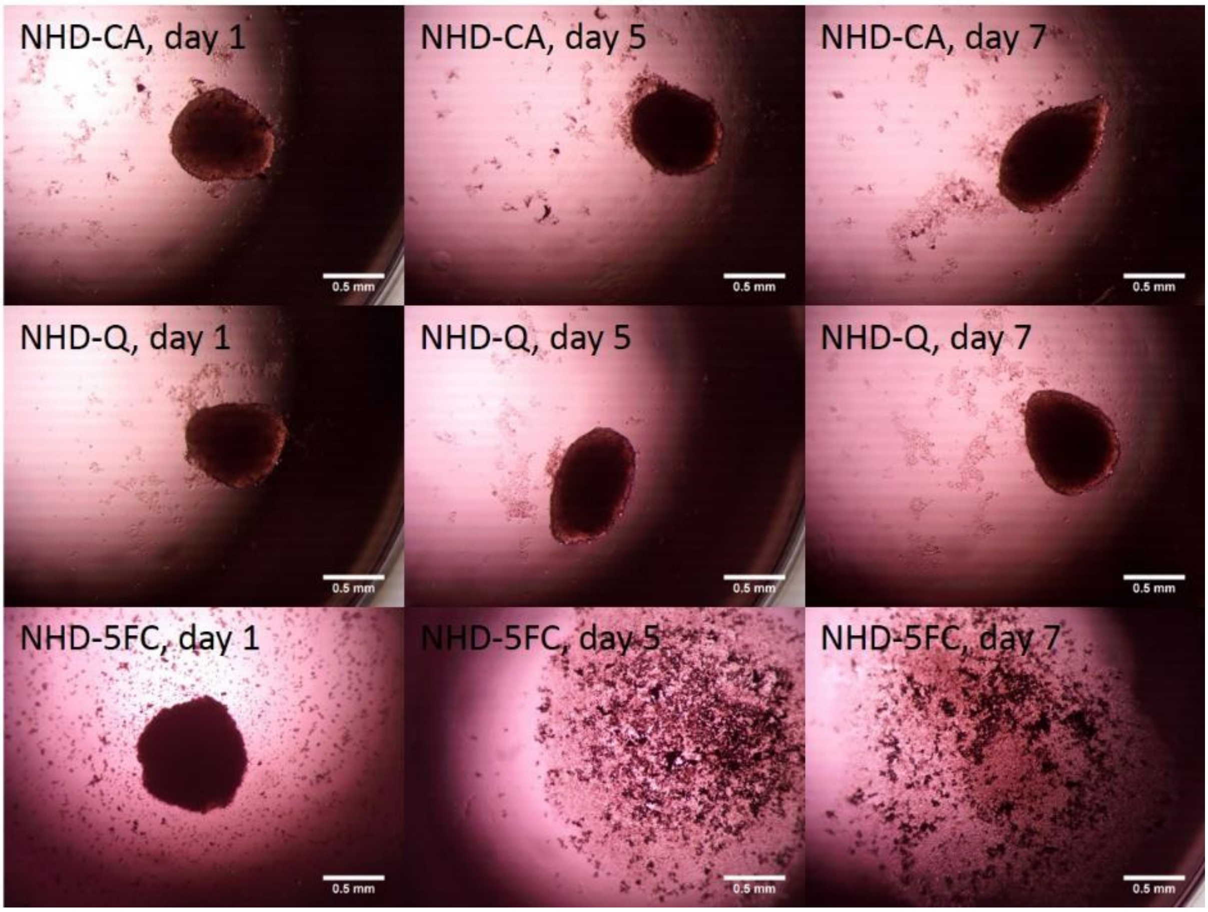

Figure S21: Microscope images of the MCF-10 A MCS treated with NHD-CA (first row), NHD-Q (second row) and NHD-5FC (third row) irradiated with 5 single doses of 2 Gy from day 1 to 2.

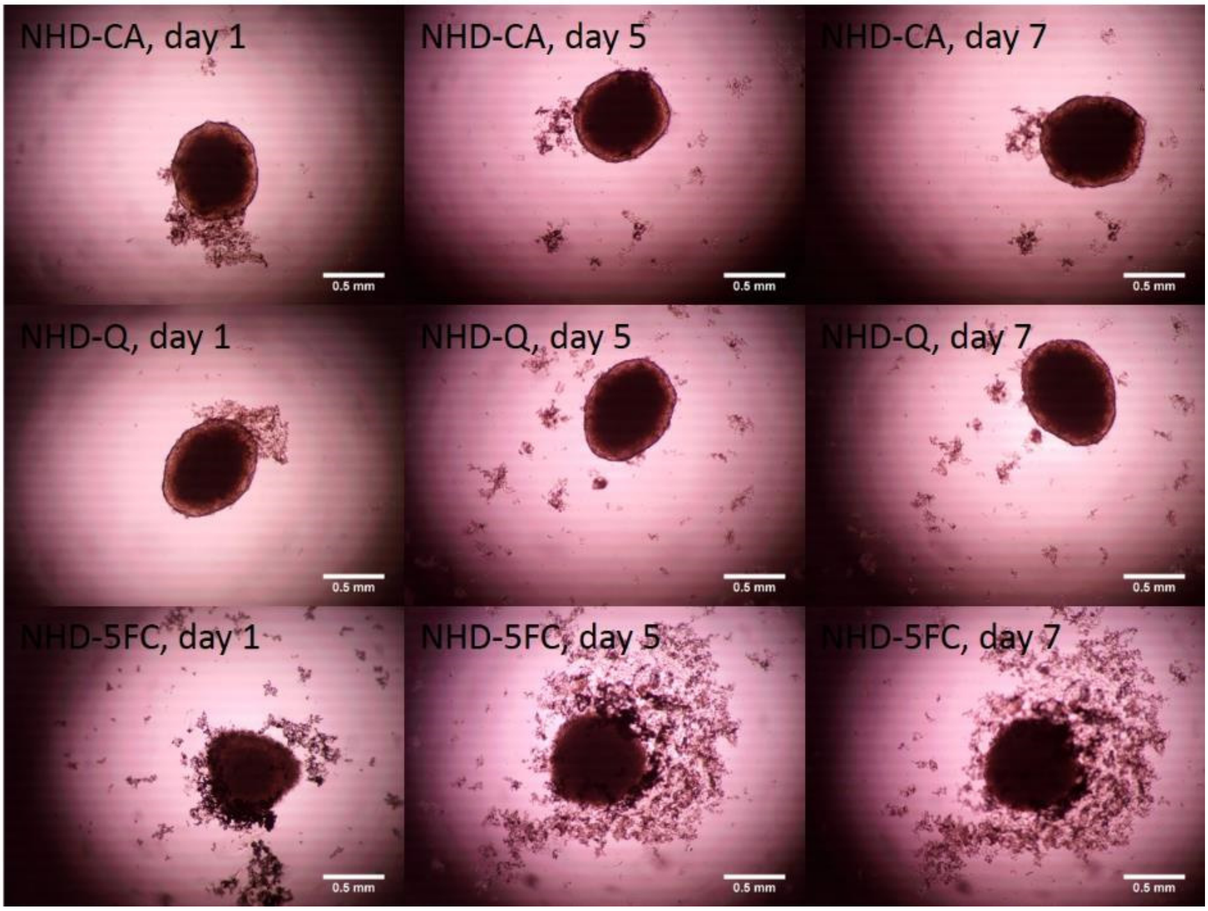

Figure S22: Microscope images of the MCF-10 A MCS treated with NHD-CA (first row), NHD-Q (second row) and NHD-5FC (third row) irradiated with 5 single doses of 2 Gy from day 1 to 5.
